# Correction of frameshift mutations in the *atpB* gene by translational recoding in chloroplasts of *Oenothera* and tobacco

**DOI:** 10.1093/plcell/koab050

**Published:** 2021-02-09

**Authors:** Irina Malinova, Arkadiusz Zupok, Amid Massouh, Mark Aurel Schöttler, Etienne H Meyer, Liliya Yaneva-Roder, Witold Szymanski, Margit Rößner, Stephanie Ruf, Ralph Bock, Stephan Greiner

**Affiliations:** Department Organelle Biology, Biotechnology and Molecular Ecophysiology, Max Planck Institute of Molecular Plant Physiology, 14476 Potsdam-Golm, Germany

## Abstract

Translational recoding, also known as ribosomal frameshifting, is a process that causes ribosome slippage along the messenger RNA, thereby changing the amino acid sequence of the synthesized protein. Whether the chloroplast employs recoding is unknown. I-iota, a plastome mutant of *Oenothera* (evening primrose), carries a single adenine insertion in an oligoA stretch [11A] of the *atpB* coding region (encoding the β-subunit of the ATP synthase). The mutation is expected to cause synthesis of a truncated, nonfunctional protein. We report that a full-length AtpB protein is detectable in I-iota leaves, suggesting operation of a recoding mechanism. To characterize the phenomenon, we generated transplastomic tobacco lines in which the *atpB* reading frame was altered by insertions or deletions in the oligoA motif. We observed that insertion of two adenines was more efficiently corrected than insertion of a single adenine, or deletion of one or two adenines. We further show that homopolymeric composition of the oligoA stretch is essential for recoding, as an additional replacement of AAA lysine codon by AAG resulted in an albino phenotype. Our work provides evidence for the operation of translational recoding in chloroplasts. Recoding enables correction of frameshift mutations and can restore photoautotrophic growth in the presence of a mutation that otherwise would be lethal.

## Teaser

Analysis of spontaneous *Oenothera* mutant and various transplastomic tobacco lines confirms operation of ribosomal frameshifting in chloroplasts.

## Introduction

Plastids (chloroplasts) are plant organelles that harbor their own genome (plastome) and are essential for many metabolic pathways, including photosynthesis and *de novo* synthesis of amino acids, nucleotides, and fatty acids (Jarvis and López-Juez, 2013). As a result of endosymbiosis, plastids evolved from a photosynthetic cyanobacterium that was engulfed by a eukaryotic cell (Herrmann, 1997; Bock, 2017). During subsequent coevolution of chloroplast and nuclear genomes, many plastid-encoded genes were lost or transferred to the nucleus (Timmis et al., 2004). Therefore, extant plastid genomes are small and contain coding information for only approximately 120 genes in green plants (Bock, 2007; Barkan, 2011). The majority of these plastid-encoded genes are crucial for plant viability and required for photoautotrophic growth, including, for example, genes encoding the large subunit of RuBisCO, the reaction center subunits of the two photosystems, and subunits of the cytochrome *b_6_f* complex and the ATP synthase. Other plastid-encoded gene products comprise components of the chloroplast gene expression machinery, such as the plastid-encoded bacterial-type RNA polymerase, ribosomal RNAs, all transfer RNAs (tRNAs), and approximately one-third of the ribosomal proteins (Zoschke and Bock, 2018). Due to its cyanobacterial ancestry, the chloroplast gene expression machinery is largely of the bacterial type (Peled-Zehavi, 2007; Zoschke and Bock, 2018).

**Figure:**
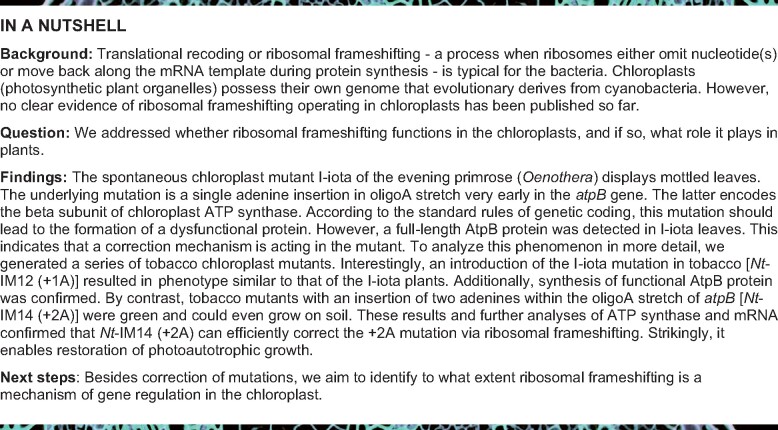


Precise genetic decoding is crucial to ensure the correct amino acid sequence in expressed proteins. During translation, spontaneous frameshifting is very rare, with a frequency of about 10^−4^ to 10^−5^ per codon (Parker, 1989; Kurland, 1992). However, evidence has accumulated for the nonuniversality and plasticity of genetic decoding in many systems (Baranov et al., 2015; Atkins et al., 2016). Two major types of alternative genetic decoding can be distinguished: global codon reassignment and local transcriptional and translational recoding (Baranov et al., 2015). Global recoding leads to deviations from the standard genetic code. For example, in some bacteria, the stop codon UGA is reassigned to code for tryptophan (Yamao et al., 1985; McCutcheon et al., 2009) or glycine (Ivanova et al., 2014). Genetic code reassignment was also detected in plastids of the parasitic plant genus *Balanophora*, where UAG is reassigned from a stop codon to tryptophan (Su et al., 2019). At the local level, alternative genetic decoding can be caused by mRNA editing, transcriptional slippage or ribosomal frameshifting (Gesteland et al., 1992).

Transcriptional slippage or RNA polymerase stuttering is a process, by which the nascent transcript or transcriptional complex slips along the DNA template, resulting in the incorporation of additional or fewer nucleotides than encoded by the DNA template (Anikin et al., 2010). Transcriptional slippage mostly affects transcription of longer homopolymeric nucleotide tracts (Baranov et al., 2005) and results in the synthesis of a heterogeneous mRNA population. The slippage can occur during all phases of the transcription cycle (Anikin et al., 2010). RNA polymerase stuttering is frequently seen in some groups of RNA viruses, including paramyxoviruses (Jacques et al., 1994), the Ebola virus (Volchkov et al., 1995), the hepatitis C virus (Ratinier et al., 2008), and plants viruses of the *Potyviridae* family (Olspert et al., 2015). Here, it often leads to the synthesis of more than one functional gene product from a single open reading frame (Volchkov et al., 1995; Hausmann et al., 1999). In a few cases, it also has been shown to compensate for a frameshift mutation in the coding region (Baranov et al., 2005). For example, a single nucleotide deletion in the human apolipoprotein B (*apoB*) gene causes hypobetalipoproteinemia. However, the mutant allele is not a null allele: in addition to the expected truncated protein, some functional full-length ApoB protein was detected that presumably was synthesized by transcriptional slippage (Linton et al., 1992).

Recoding at the level of translation can occur by programmed ribosomal frameshifting (PRF), a process in which the ribosome changes the frame it translates (Ketteler, 2012; Baranov et al., 2015; Atkins et al., 2016). PRF can occur in the forward or reverse direction (relative to the 0-frame) as a consequence of the ribosome either skipping one nucleotide (+1) or slipping back one nucleotide (−1) (Ketteler, 2012). This can result in the production of two (or more) proteins from the same mRNA (Caliskan et al., 2017). Various signals are known to affect the efficiency of ribosomal frameshifting such as the presence of a Shine-Dalgarno-like sequence upstream of the slip site and the mRNA secondary structure downstream of the slip site (Baranov et al., 2006). −1 frameshifting is more common than +1 frameshifting and, therefore, −1 PRF has been analyzed in more detail (Belew and Dinman, 2015). −1 ribosomal frameshifting requires the slippery sequence motif X_XXY_YYZ, where X denotes any three identical nucleotides, Y denotes A or U, and Z is A, U, or C (Brierley et al., 1992). The P- and A-sites of the tRNA anticodon re-pair from XXY to XXX and YYZ to YYY, respectively (Jacks et al., 1988). In agreement with the consensus sequence, the slip site can be homopolymeric such as the A_AAA_AAA site in the adenomatous polyposis coli tumor suppressor protein of some nematodes (Baranov et al., 2011). The best-described bacterial −1 frameshift has the type A_AA.A_AA.G (with the codons of the original frame being separated by an underlined space, and the codons in the shifted frame by a dot) (Baranov et al., 2006). A pseudoknot in the mRNA structure is another motif that is often involved in successful ribosomal frameshifting. The pseudoknot consists of two or more stem-loop motifs and resides 3′ of the slippery site, separated from it by a spacer region of 5–9 nt (Ketteler, 2012). Together, slippery sequence and RNA secondary structures enable pausing of the ribosome and eventually frameshifting (Namy et al., 2006).

While the mechanisms of transcriptional slippage and translational frameshifting are distinct, both can occur on similar sites (Atkins et al., 2016). In *Escherichia coli*, synthesis of functionally distinct DNA polymerase III subunits (τ and γ) is achieved by ribosomal frameshifting, whereas in *Thermus thermophiles*, this is the result of transcriptional slippage followed by standard translation of the multiple mRNAs (Larsen et al., 2000). It is sometimes difficult to distinguish between low levels of transcriptional slippage and ribosomal frameshifting (Atkins et al., 2016). For example, recoding in the core protein of the hepatitis C virus was first ascribed to PRF, but later attributed to transcriptional slippage (Ratinier et al., 2008).

Ribosomal frameshifting in plants has been shown to occur on some viral RNAs (Miller and Giedroc, 2010; Atkins et al., 2016). Recently, it was reported that a conserved peptide upstream open reading frame in the mRNA of *AT3G57170* is a possible candidate for ribosomal frameshifting in *Arabidopsis* (van der Horst et al., 2019). Furthermore, ribosomal frameshifting was detected *in vitro* using wheat-germ extract (Napthine et al., 2003). Finally, indirect evidence for PRF was reported in chloroplasts (Kohl and Bock, 2009). By generating transplastomic tobacco plants, it was shown that the bacterial IS150 transposon was mobilized inside the chloroplast, which results in the accumulation of transposition intermediates. Since the synthesis of IS150 transposase requires PRF (Polard et al., 1991; Vögele et al., 1991), PRF was proposed to exist in plastids.

Mutants in the chloroplast genome have been proven as a powerful tool in the analysis of chloroplast gene expression and photosynthesis (Schaffner et al., 1995; Landau et al., 2009; Greiner, 2012). Based on their chlorotic phenotypes, a collection of 51 spontaneous chloroplast mutants was isolated in evening primroses (*Oenothera*) (Kutzelnigg and Stubbe, 1974; Stubbe and Herrmann, 1982). Recently, this collection was systematically characterized by full plastome sequencing (Massouh et al., 2016). Chloroplast mutants affecting all photosynthetic complexes as well as the chloroplast translation machinery were identified. This collection also offers great potential to uncover novel mechanisms of chloroplast gene regulation (Greiner, 2012; Massouh et al., 2016). One of the most striking mutant phenotypes observed in this collection was the mottled phenotype of the I-iota mutant. It displays green spots on white leaves which grow with age, leading to a nearly green phenotype (Figure 1C in Massouh et al., 2016). The underlying mutation is a single adenine insertion (+1A) located within an oligoA stretch in the *atpB* gene, encoding the β-subunit of the plastid ATP synthase (Massouh et al., 2016). The AtpB protein is part of the α_3_β_3_ catalytic center harboring the nucleotide binding sites of the enzyme (Hahn et al., 2018). Here, we have analyzed the physiological and biochemical consequences of the +1A mutation in the I-iota mutant. We provide evidence for partial rescue of AtpB synthesis by ribosomal frameshifting. To characterize the recoding mechanism, a set of transplastomic tobacco mutants with mutated frameshift sites were generated and analyzed. Recoding efficiency was further assessed using a luciferase-based reporter system in *E. coli*. We present clear evidence for recoding in the chloroplasts depending on length and composition of the slip site and discuss its functional implications.

**Figure 1:**
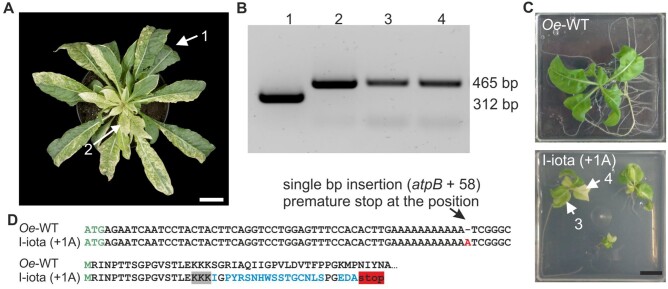
Phenotype and genotype of the I-iota mutant. **(A)** Variegated *Oenothera* plant at the mature rosette stage (seven weeks old) carrying homoplasmic sectors containing the nursery plastome (green) [1] and homoplasmic sectors with I-iota chloroplasts (mottled) [2]. Scale bar: 5 cm. **(B)** PCR confirming homoplasmy of mottled and green sectors (1: nursery plastome [312 bp], 2-4: I-iota plastome [465 bp]; note the absence of the 312 bp band in lanes 2–4). **(C)** Phenotype of wild-type (*Oe*-WT) and I-iota homoplasmic plants cultivated under mixotrophic conditions in sterile culture. Lanes 3 and 4 in panel B confirm homoplasmy of the mottled plants. Scale bar: 1 cm. **(D)** 5′ part of the *atpB* gene of *Oe*-WT and I-iota (+1A). Two sequences on the top are genomic DNA sequences, two sequences on the bottom are amino acid sequences based on standard rules of genetic decoding. The mutation at position +58 in an oligoA stretch of *atpB* in I-iota is denoted in red. The lysine stretch affected by the mutation is highlighted in grey. Green letters indicate the start codon. Blue letters indicate amino acids expected to deviate from the wild-type sequence according to the standard rules of genetic decoding.

## Results

### Molecular and biochemical characterization of the *Oenothera* I-iota mutant

I-iota was initially identified as chloroplast mutant displaying a mottled phenotype (Sears and Herrmann, 1985; Massouh et al., 2016). When the mutation is present in the homoplasmic stage, the plants cannot grow photoautotrophically. Therefore, to facilitate growth in soil, plants must be kept as variegated plants that are heteroplasmic and contain green nursery tissue (with wild-type chloroplasts) and pale sectors with mutated chloroplasts (Figure 1A). Taking advantage of biparental chloroplast inheritance in evening primrose, appropriate plants can easily be generated by a simple cross. To this end, a maintainer line with the chloroplast genome of *Oenothera parviflora* is crossed to the I-iota mutant originating from *O. elata* (cf. Greiner, 2012; Massouh et al., 2016; see “Materials and methods” section). To confirm that the mosaic pattern of I-iota was not caused by incomplete vegetative sorting of the nursery plastome in the mutated I-iota sectors (Figure 1A; cf. Greiner, 2012), tests for homoplasmy in green and mottled tissue were performed. The tests took advantage of a length polymorphism in the plastid *clpP* gene that results in a larger PCR amplification product in the I-iota plastome of *O. elata* compared to the nursery plastome from *O. parviflora* (Greiner et al., 2008). A single fragment of 312 bp was detected in green parts of the plant, whereas a product of 465 bp was identified in mottled areas (Figures 1A and 1B), thus confirming the homoplasmic state of the I-iota mutation in the mottled leaf sectors. To further characterize the phenotype of the I-iota mutant, homoplasmic I-iota plants were grown under mixotrophic conditions in sterile culture with sugar supplementation. Compared to wild-type plants, homoplasmic I-iota mutants were strongly reduced in growth and displayed a pale or variegated phenotype (Figure 1C). Homoplasmy of the I-iota mutant in sterile culture was verified by PCR using the same marker as described above and confirmed for both the pale and green parts of the leaves (Figure 1B, lanes 3 and 4). All samples referred to as I-iota later in the text were tested for homopasmy by PCR before further analysis.

Even without the knowledge of the underlying mutation, a defect in the chloroplast ATP synthase was predicted for I-iota. Moreover, the AtpB subunit was correctly identified as targeted by the mutation (Herrmann et al., 1980; Sears and Herrmann, 1985). Indeed, mass spectrometry analysis confirmed reduced amounts of AtpA, AtpB, and AtpE in I-iota tissue compared to the wild type (Figure 2A). However, Sears and Herrmann (1985) observed an unexpected high molecular mass band using their anti-AtpE antibody. Furthermore, translational coupling of *atpB* and *atpE* was predicted (Zurawski et al., 1982; Gatenby et al., 1989). In the present immunoblot analysis AtpE bands with the expected size of 14.5 kDa were identified in I-iota tissue (Figure 2B) and no unspecific high molecular mass band was detected in I-iota tissue (Supplemental Figure 1A), indicating the absence of an AtpB/AtpE fusion product as suggested by Sears and Herrmann (1985). This is in accordance with *atpB* and *atpE* translational independency detected previously in ribosome footprint profiling experiments (Zoschke et al., 2013). Recently, a single adenine insertion (+1A) in *atpB* was identified as the only mutation in the I-iota plastome (Massouh et al., 2016). The mutation is located within an oligoA stretch near the 5′-end of the gene (Figure 1D). The identified mutation results in a premature stop codon and formation of a truncated protein (41 amino acids long; Figure 1D and Table 1). Confirming the previous report of Sears and Herrmann (1985), the full-length AtpB protein was detected in I-iota (+1A) leaf tissue by immunoblot analysis (Figure 2B). Importantly, although the AtpB protein level was lower in I-iota (+1A) leaves than in the wild type (*Oe*-WT), no difference in the mobility of the protein band between the wild type and the I-iota was observed (Supplemental Figure 1A). Mature leaves of I-iota contained higher levels of AtpB than young leaves (Figure 2B), suggesting that functional AtpB accumulates over time. This observation can also explain the age-dependent greening phenotype of I-iota mutant plants (Massouh et al., 2016).

**Figure 2:**
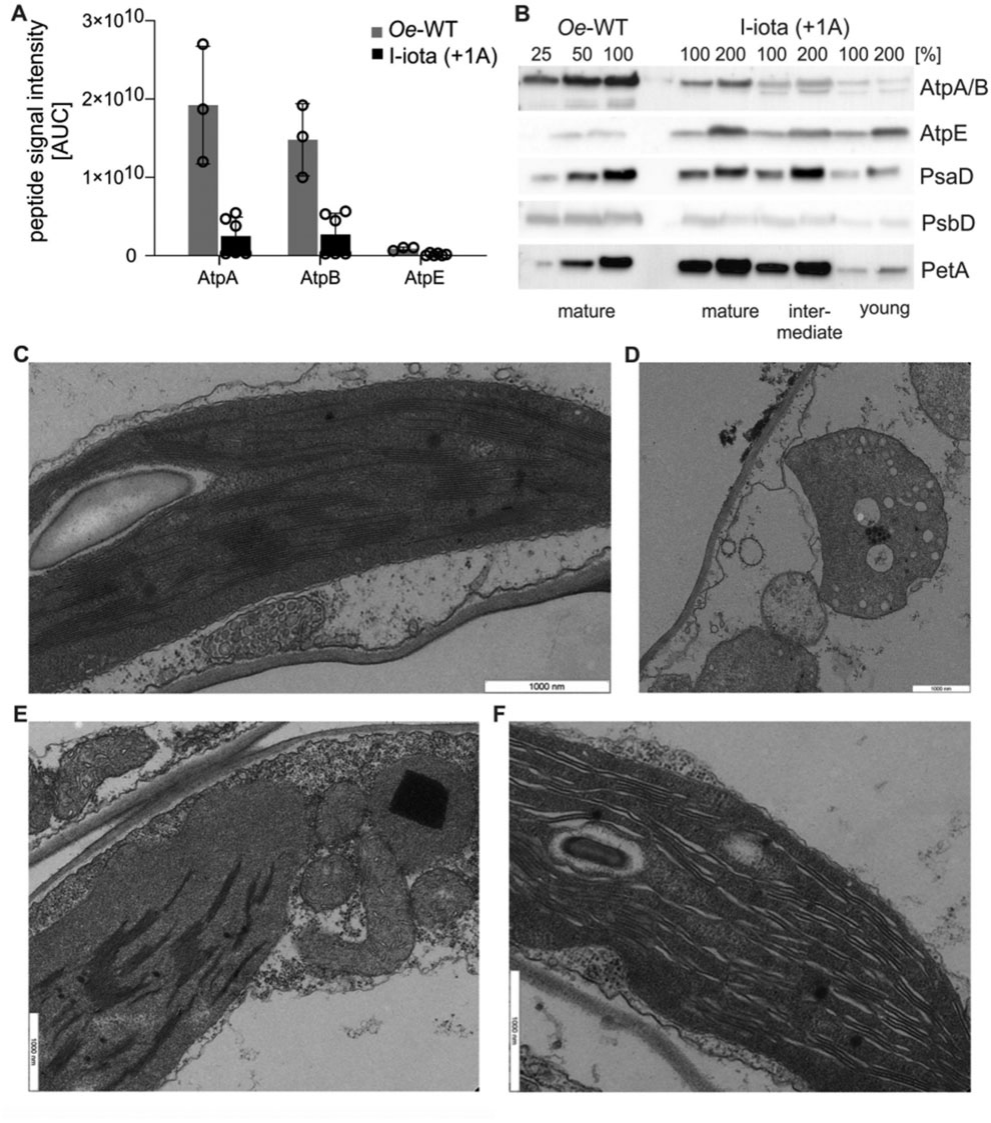
Analysis of ATP synthase and chloroplast ultrastructure in the I-iota (+1A) mutant and the wild type (*Oe*-WT). **(A)** MS quantification of ATP synthase subunits in wild-type and I-iota leaves. Thylakoids isolated from three *Oe*-WT and six I-iota plants were subjected to analysis. Columns display average data and standard deviation. Data points are shown. Signal intensities of peptides (AUC: area under the curve) of analyzed protein subunits, were normalized to BSA, which was used as internal control. **(B)** Immunoblot detection of ATP synthase units (AtpA/B and AtpE) and photosynthetic subunits of PSI (PsaD), PSII (PsbD) and Cyt *b*_6_*f* (PetA) from crude extracts of the wild-type (*Oe*-WT) and I-iota leaves. For the I-iota mutant, leaf tissues of different ages were analyzed (young: top leaves of the rosette, intermediate: mottled leaves from the middle of the rosette, mature: greenish part of leaves from the base of the rosette; see Figure 1A). Leaf tissues were tested for homoplasmy using the *clpP* marker prior to further analysis (Supplemental Figure 1C). Equal fresh weight (20 mg, for details see “Materials and methods” section) were used for crude extract preparation. Samples were loaded based on equal sample volume (20 µL = 100%). The experiments were repeated at least once, displaying similar tendency. C–F, TEM analysis of wild-type (*Oe-*WT) and I-iota (+1A) leaf tissue. Seven-week-old plants were investigated. **(C)** Wild-type chloroplast. D–F, I-iota (+1A) chloroplasts. **(D)** Proplastid-like chloroplast. **(E)** Chloroplast with developing thylakoid membrane. **(F)** Mature wild-type-like chloroplast.

**Table 1:** Possibility space of correction by recoding in *Oenothera* I-iota and tobacco transplastomic lines

Species	Genotype	Correction	5′ amino acid sequence of AtpB
*Oenothera*	O*e*-WT	−	MRINPTTSGPGVSTLE **KKK** -SGRIAQIIGPVLDVTFPPGKMPNIYNALVVKG[…]
	I-iota (+1A)	−	MRINPTTSGPGVSTLE **KKK** - * I * G * PYRSNHWSSTGCNLS * PG * EDA * **stop**
		+1	MRINPTTSGPGVSTLE **KKK** -SGRIAQIIGPVLDVTFPPGKMPNIYNALVVKG[…]
		+4	MRINPTTSGPGVSTLE **KK** --SGRIAQIIGPVLDVTFPPGKMPNIYNALVVKG[…]
		−2	MRINPTTSGPGVSTLE **KKKK** SGRIAQIIGPVLDVTFPPGKMPNIYNALVVKG[…]
Tobacco	*Nt-*WT	−	MRINPTTSGSGVSTLE **KK** -NPGRVVQIIGPVLDVAFPPGKMPNIYNALVVQG[…]
	*aadA-atpB*	−	MRINPTTSGSGVSTLE **KK** -NPGRVVQIIGPVLDVAFPPGKMPNIYNALVVQG[…]
	*Nt*-IM11 (AAG)	−	MRINPTTSGSGVSTLE **KK** -NPGRVVQIIGPVLDVAFPPGKMPNIYNALVVQG[…]
	*Nt*-IM12 (+1A)	−	MRINPTTSGSGVSTLE **KKK** * PGACRPNHRSGTRCSLSPGQDAEYL * **stop**
		+1	MRINPTTSGSGVSTLE **KK** -NPGRVVQIIGPVLDVAFPPGKMPNIYNALVVQG[…]
		+4	MRINPTTSGSGVSTLE **K** —-NPGRVVQIIGPVLDVAFPPGKMPNIYNALVVQG[…]
		−2	MRINPTTSGSGVSTLE **KKK** NPGRVVQIIGPVLDVAFPPGKMPNIYNALVVQG[…]
	*Nt*-IM13 (AAG + 1A)	−	MRINPTTSGSGVSTLE **KKK** * PGACRPNHRSGTRCSLSPGQDAEYL * **stop**
		+1	MRINPTTSGSGVSTLE **KK** -NPGRVVQIIGPVLDVAFPPGKMPNIYNALVVQG[…]
		+4	MRINPTTSGSGVSTLE **K** —-NPGRVVQIIGPVLDVAFPPGKMPNIYNALVVQG[…]
		−2	MRINPTTSGSGVSTLE **KKK** NPGRVVQIIGPVLDVAFPPGKMPNIYNALVVQG[…]
	*Nt*-IM14 (+2A)	−	MRINPTTSGSGVSTLE **KKK** * TRGVSSKSSVRY * **stop**
		+2	MRINPTTSGSGVSTLE **KK** -NPGRVVQIIGPVLDVAFPPGKMPNIYNALVVQG[…]
		−1	MRINPTTSGSGVSTLE **KKK** NPGRVVQIIGPVLDVAFPPGKMPNIYNALVVQG[…]
	*Nt*-IM16 (−1A)	−	MRINPTTSGSGVSTLE **KK** * TRGVSSKSSVRY * **stop**
		−1	MRINPTTSGSGVSTLE **KK** -NPGRVVQIIGPVLDVAFPPGKMPNIYNALVVQG[…]
		−4	MRINPTTSGSGVSTLE **KKK** NPGRVVQIIGPVLDVAFPPGKMPNIYNALVVQG[…]
	*Nt*-IM18 (−2A)	−	MRINPTTSGSGVSTLE **KK** * PGACRPNHRSGTRCSLSPGQDAEYL * **stop**
		−2	MRINPTTSGSGVSTLE **KK** -NPGRVVQIIGPVLDVAFPPGKMPNIYNALVVQG[…]
		+1	MRINPTTSGSGVSTLE **K** —-NPGRVVQIIGPVLDVAFPPGKMPNIYNALVVQG[…]

The lysine stretch affected by the mutation is in bold. Underlined italics indicate amino acids expected to deviate from the wild-type sequence according to the standard rules of genetic decoding. Premature stop codons are indicated “−” denotes no correction mechanism, “+N” and “−N” indicate the direction of translational recoding (for details see “Introduction” section).

Analysis of plastid ultrastructure by transmission electron microscopy confirmed the age-dependent developmental gradient in thylakoid membrane formation of I-iota (Figures 2D to 2F). Although mature leaves of I-iota still had slightly disorganized thylakoid membranes, they were similar to wild-type thylakoids (Figures 2C versus 2F). Furthermore, the abundance of photosynthetic subunits was assessed (Figure 2B). Young white leaves of I-iota mutant displayed a major reduction in the nucleus-encoded stromal ridge subunit of PSI PsaD, the chloroplast-encoded PSII reaction center subunit PsbD, and the plastid-encoded cytochrome-*f* subunit of the cytochrome *b_6_f* complex, PetA. Mature I-iota mature leaves still showed a reduction in PsaD and PsbD compared to the wild type. Interestingly, no difference in PetA abundance was detected between *Oe-*WT and I-iota mature leaves. The presence of a full-length AtpB protein in I-iota tissue was verified by in-gel tryptic digestion followed by liquid chromatography-mass spectrometry (LC–MS/MS; Table 2).

**Table 2:** Identification of AtpB in *O*e*nothera* I-iota and transplastomic tobacco lines and the corresponding wild type and control lines by in-gel tryptic digestion followed by LC–MS/MS analysis

Genotype	No. of significant matches	No. of significant sequences
*Oe*-WT	556	26
I-iota (+1A)	113	14
*Nt*-WT	249	34
*aadA-atpB*	77	24
*Nt*-IM11 (AAG)	155	31
*Nt*-IM12 (+1A)	28	13
*Nt*-IM13 (AAG+1A)	n.d.	n.d.
*Nt*-IM14 (+2A)	23	19
*Nt*-IM16 (−1A)	10	8
*Nt*-IM18 (−2A)	6	5

At least three samples derived from different plants were subjected to the LC–MS/MS with similar results. Data were analyzed using MASCOT MS/MS Ion Search with default parameters and significance threshold *P* < 0.05. n.d., not detected.

Finally, as *in vivo* measure of chloroplast ATP synthase activity per thylakoid membrane, the proton conductivity of the thylakoid membrane (gH^+^) was assessed by decay kinetics of the electrochromic shift signal (ECS) during a short interval of darkness. In mature leaves of I-iota, a residual ATP synthase activity of 29% compared to the wild type was detectable (Figure 3A), confirming that the chloroplast ATP synthase is indeed active in the I-iota mutant. As a consequence of the reduced ATP synthase activity, the entire photosynthetic apparatus of I-iota was affected. The chlorophyll content per leaf area was strongly reduced to 24% of the wild-type level (Table 3) and, assuming that this reduction is roughly proportional to the general loss of thylakoids per leaf area, ATP synthase activity per area would be decreased to less than 10% of that of wild-type plants. In good agreement with this severe loss of ATP synthase activity per leaf area, light response curves of chlorophyll-*a* fluorescence parameters revealed that linear electron transport per leaf area (calculated from PSII yield measurements) was strongly decreased in I-iota, and the acceptor side of PSII (here shown as the chlorophyll-*a* fluorescence parameter qL) became more rapidly reduced (Figure 3B). The strong decrease of the chlorophyll *a/b* ratio is in line with the reduced accumulation of the photosystems, which was shown by immunoblots against PsaD and PsbD (Figure 2B, see above). The photosynthetic reaction centers exclusively bind chlorophyll *a*, while the antenna proteins bind both chlorophyll *a* and *b* and therefore likely were less severely affected than the reaction centers (Table 3). Also, F_V_/F_M_, the maximum quantum efficiency of photosystem II (PSII) in the dark-adapted state, was clearly reduced in I-iota, pointing to PSII photoinhibition and the presence of uncoupled antenna proteins. All these changes bear close similarity to photosynthetic defects previously reported for tobacco and maize (*Zea mays*) mutants with strongly diminished accumulation of ATP synthase (Rott et al., 2011; Zoschke et al., 2012), and can be ascribed to an increased proton motive force across the thylakoid membrane (because its consumption by ATP synthase is massively slowed down).

**Figure 3:**
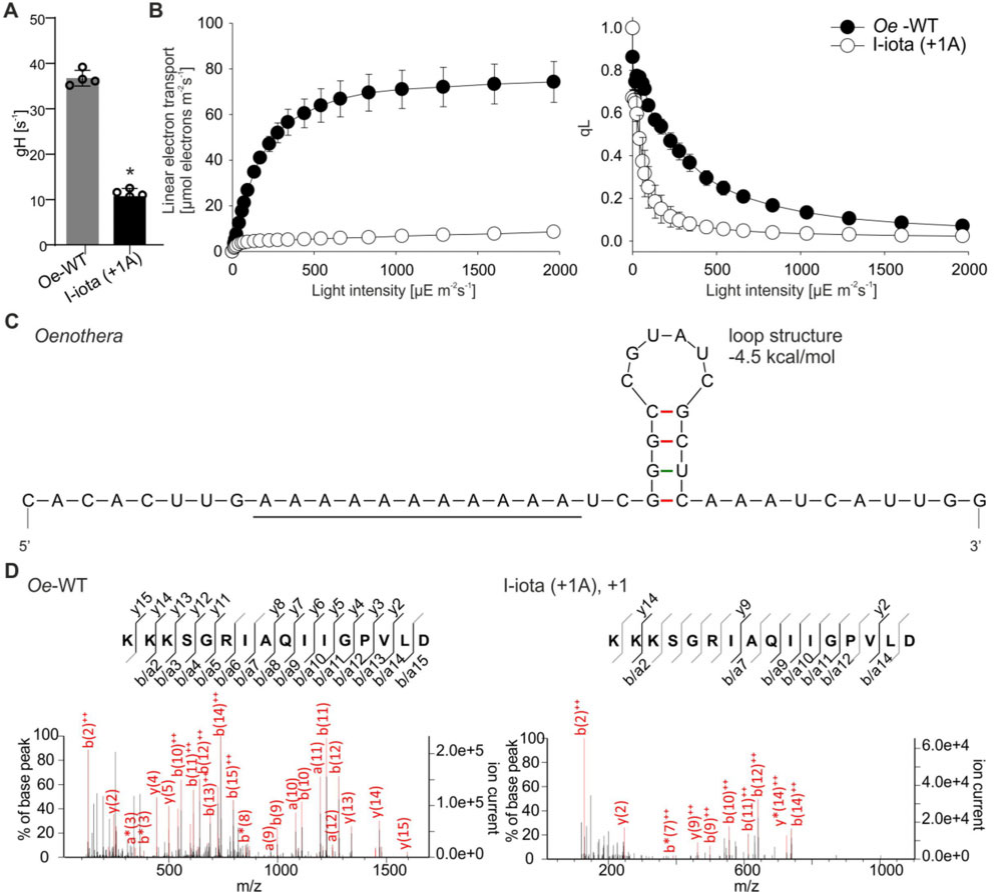
Photosynthetic parameters and analysis of target peptides in the I-iota mutant and the wild type (*Oe*-WT). **(A)** ATP synthase activity per thylakoid membrane determined from dark-interval decay kinetics of the proton motive force-related electrochromic shift signal. Columns display average data and standard deviation. Data points for the individual *Oe*-WT and I-iota plants measured are shown in addition to the average values. Mature leaves of four *Oe*-WT plants and five I-iota plants were subjected to the analysis. The asterisk indicates a significant difference compared to the wild type (*t* test, *P* < 0.01, Supplemental Data Set 1). **(B)** Light–response curves of linear electron flux (left panel) and chlorophyll-*a* fluorescence parameter qL (a measure of the redox state of the PSII acceptor side; right panel) in the wild type **(***Oe*-WT**)** and I-iota. Eight wild-type plants and 10 mutant plants were measured. Error bars represent the standard deviation. **(C)** Predicted mRNA secondary structure of *atpB* near the slip site (oligoA stretch; underlined). **(D)** LC–MS/MS fragmentation spectrum of the targeted AtpB peptide. +1 denotes the direction of translational recoding (for detail see “Introduction” section). The KKK stretch encoded at the slip site is identified as “b−ions” and “y−ions” type fragment ions.

**Table 3:** Analysis of photosynthetic parameters

Growth conditions	Genotype	Chlorophyll (mg m^−2^)	Chlorophyll *a/b*	F_V_/F_M_
Greenhouse	*Oe*-WT	570.3 ± 12.8^**a**^	3.6 ± 0.1^**a**^	0.79 ± 0.01^**a**^
	I-iota (+1A)	137.5 ± 51.2^**b**^	2.7 ± 0.2^**b**^	0.64 ± 0.02^**b**^
	*Nt*-WT	438.4 ± 36.0^**c**^	4.0 ± 0.2^**c**^	0.83 ± 0.01^**a**^
	*aadA-atpB*	414.7 ± 38.5^**c**^	3.9 ± 0.1^**ac**^	0.81 ± 0.02^**a**^
	*Nt*-IM11(AAG)	451.0 ± 23.5^**c**^	3.8 ± 0.2^**ac**^	0.83 ± 0.01^**a**^
	*Nt*-IM14 (+2A)	128.5 ± 17.0^**b**^	2.6 ± 0.2^**b**^	0.53 ± 0.08^**c**^
Sterile culture	*Nt-*WT	297.3 ± 87.0^**ab**^	3.50 ± 0.24^**a**^	0.79 ± 0.04^**a**^
	*aadA-atpB*	262.8 ± 47. 9^**a**^	3.42 ± 0.13^**a**^	0.77 ± 0.03^**a**^
	*Nt*-IM11 (AAG)	257.2 ± 63.1^a^	3.50 ± 0.12^**a**^	0.79 ± 0.03^**a**^
	*Nt*-IM12 (+1A)	243.5 ± 70.1^a^	2.34 ± 0.27^**b**^	0.33 ± 0.14^**b**^
	*Nt*-IM14 (+2A)	372.9 ± 78.1^b^	2.78 ± 0.21^**c**^	0.64 ± 0.18^**c**^
	*Nt*-IM16 (−1A)	240.4 ± 95.7^**a**^	2.17 ± 0.36^**b**^	0.44 ± 0.23^**b**^

Three to five plants were analyzed for both wild-types (*Oe*-WT and *Nt*-WT), the I-iota (+1) mutant, and the generated transplastomic tobacco lines. For each construct, at least two independent transplastomic lines were analyzed. Because no significant differences between these lines were observed, average data ± sd are shown for each genotype. Letters indicate samples that were not significantly different according to one-way ANOVA with Holm–Sidak post hoc testing (*P* < 0.05; Supplemental Data Set 1). ANOVA tests were performed separately for greenhouse- and sterile culture-grown plants

This increased proton motive force leads to a strong acidification of the thylakoid lumen, which in turn slows down linear electron transport due to “photosynthetic control” of plastoquinol re-oxidation at the cytochrome *b_6_f* complex (Nishio and Whitmarsh, 1993), and also results in the stronger reduction of the PSII acceptor side, increased photoinhibition of PSII, and loss of photosystems (Rott et al., 2011; Schöttler et al., 2015). Taken together, these data strongly suggest partial compensation of the I-iota (+1A) mutation by a recoding mechanism that operates in I-iota tissue. Interestingly, analysis of the predicted structure of the *atpB* mRNA revealed the presence of a potential slip site (oligoA stretch) and a stem-loop-type secondary structure downstream of the slip site (Figure 3C). Both motifs are known to be crucial elements for PRF. In contrast, a homopolymeric nucleotide sequence alone can be sufficient for induction of transcriptional slippage (see “Introduction” section).

Independent of the underlying molecular mechanism, mutation compensation is only partial and, therefore, will result in the formation of both in-frame and out-of-frame polypeptides (see “Introduction” section). To characterize the process of mutation correction in I-iota at the protein level, we analyzed the lysine stretch encoded by the oligoA tract in which the mutation is located (Figure 1D). Since frame correction can occur in forward or reverse direction, different scenarios are possible (Table 1): (1) uncorrected gene expression will result in formation of a truncated polypeptide; (2) +1 correction would lead to the synthesis of a protein identical to the wild type; and (3) +4 or −2 correction would result in the synthesis of proteins that differ from the wild type in the number of lysines in the stretch (four or two lysines, respectively, instead of three in the wild-type protein). Consequently, resolving the number of lysines and the amino acid sequence downstream is crucial to the understanding of the correction mechanism. Unfortunately, standard trypsin digestion cannot be applied for mass spectrometric peptide identification, because trypsin cleaves at the carboxyl site of lysine and arginine residues (Tsiatsiani and Heck, 2015), and thus, is unsuitable to resolve the number of lysines in a peptide. As an alternative, we used in-gel digestion with glutamyl peptidase I (Glu-C) that cleaves the amino acid chain at aspartate and glutamate residues (Tsiatsiani and Heck, 2015). The peptide sequence information obtained from tandem MS/MS was then analyzed against the *in silico* digested AtpB sequence from wild-type *Oenothera* and possible out-of-frame peptides that could be produced in the I-iota mutant (Table 1;Supplemental Table 1). No in-frame peptides corresponding to the expected truncated polypeptide were detected in I-iota samples, possibly suggesting that the truncated protein is unstable and condemned to rapid degradation. Instead, the same number of three lysines was detected in both the wild type and I-iota, revealing that the I-iota mutant indeed produces the wild-type AtpB protein and strongly suggesting that a +1 correction mechanism operates in the mutant chloroplasts (Figure 3D and Table 1).

### Reproduction of the I-iota mutation in transplastomic tobacco plants

Comparison of the plastid *atpB* sequences revealed that the oligoA stretch near the 5′-end of the coding region is conserved among seed plants (Figure 4A). Moreover, a similar stem–loop structure was predicted to reside downstream of the oligoA tract in the *atpB* mRNAs in different species and is also present in the cigarette tobacco (*Nicotiana tabacum*; Figure 4;Supplemental Figure 2). We, therefore, attempted to reproduce the frame-correcting mechanism in tobacco chloroplasts, an experimental system amenable to an easy genetic manipulation. To obtain insights into the sequence requirements for reading frame correction, a set of transplastomic tobacco lines was generated (Figure 5A). To mimic the *Oenothera* I-iota mutation, a single adenine was inserted into the oligoA stretch within the 5′-part of the cloned tobacco *atpB* genes, generating plastid transformation vector pIM12 (+1A). The corresponding transplastomic lines will subsequently be referred to as *Nt*-IM12 (+1A) plants. To analyze the role of the oligoA sequence in the induction of recoding, the construct pIM13 (AAG+1A) was generated, in which the oligoA stretch was disrupted by replacing adenines with guanines in the third codon position (Figure 5A). These mutations do not alter the identity of the encoded amino acids, because both AAA and AAG triplets encode lysine. A similar construct with a disrupted oligoA stretch but without the frameshift mutation was generated as control [pIM11 (AAG); Figure 5A]. All transplastomic mutants were produced by biolistic chloroplast transformation using the *aadA* cassette as selectable marker that confers resistance to spectinomycin (Svab and Maliga, 1993; Figure 6A). Insertion of the *aadA* cassette alone did not affect plant growth and chloroplast ATP synthase activity (*aadA-atpB* control plants; Figure 5B; Rott et al., 2011).

**Figure 4:**
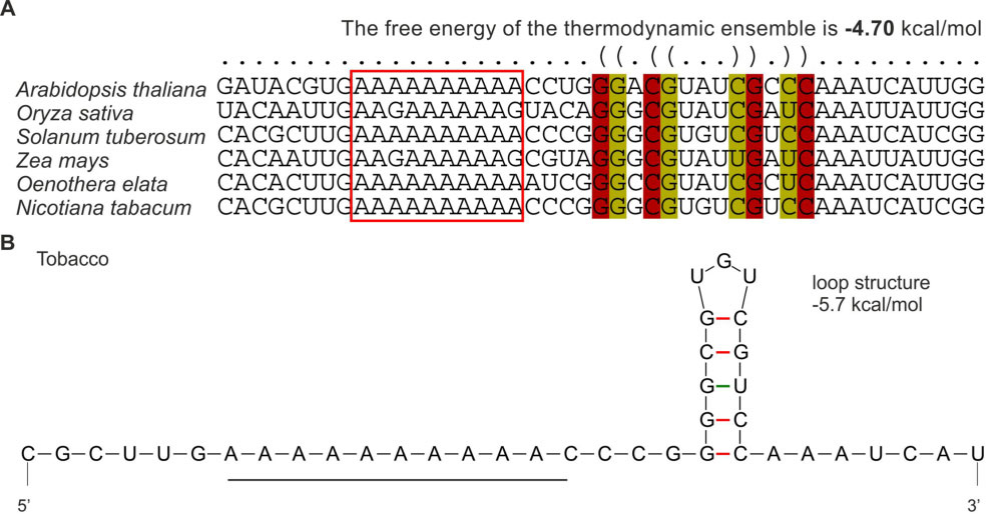
Sequence conservation and folding of the 5′-parts of the *atpB* coding region in different species. **(A)** Structure annotated alignment was performed using the RNAalifold web server. Note that all species have an oligopurine motif at the site where slippage occurs in *Oenothera*. The homopolymeric oligoA stretch (boxed in red) is conserved in *Arabidopsis thaliana*, *Solanum tuberosum*, *O. elata*, and *N. tabacum*. The characters “(“and”)” correspond to 5′ and 3′-base in the base-pare, respectively, while “.” denotes an unpaired base. **(B)** Predicted mRNA secondary structure of *atpB* near the slip site (oligoA stretch; underlined) in tobacco.

**Figure 5:**
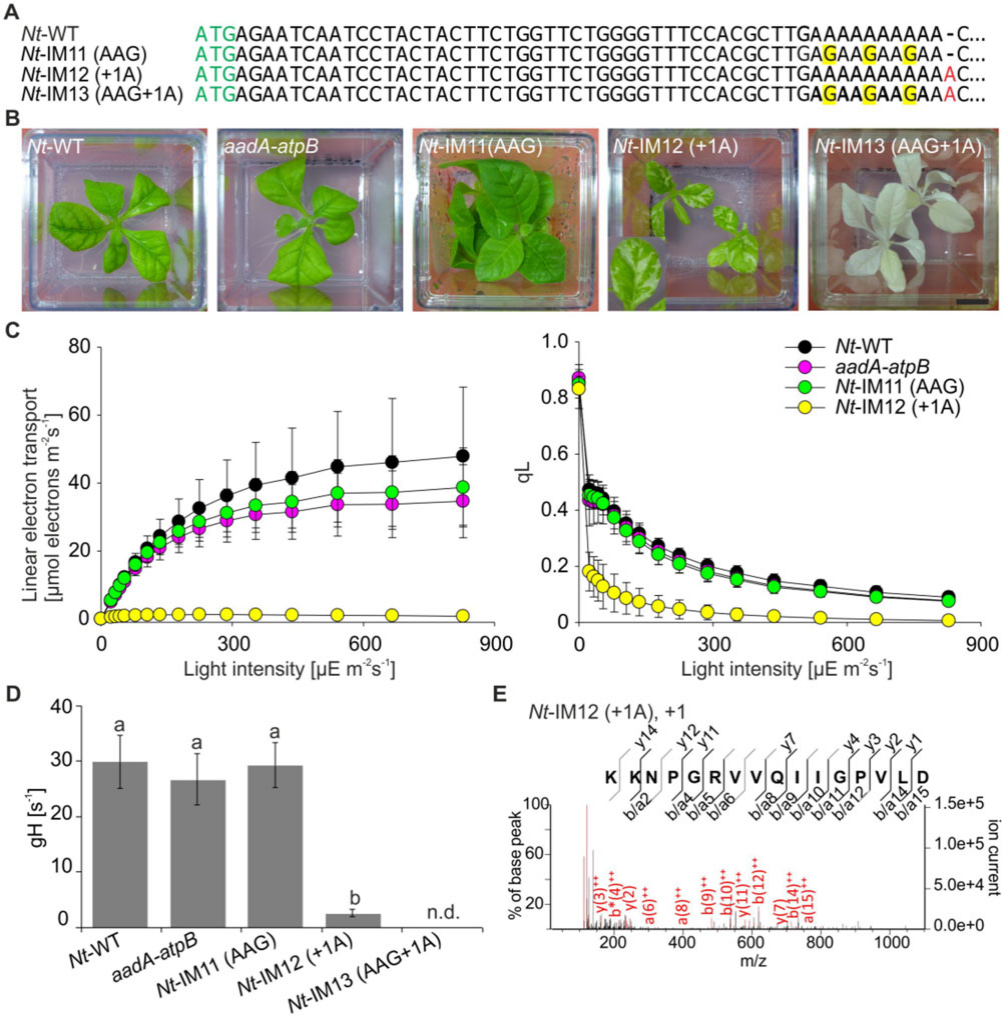
Generation and characterization of transplastomic tobacco plants carrying the I-iota mutation and/or disrupting the upstream oligoA stretch. **(A)** Mutants generated to analyze recoding in tobacco. The sequence of the 5′-part of *atpB* from the start codon (green) to the oligoA stretch are presented. Red letters represent insertions. Adenines replaced by guanines are highlighted in yellow. **(B)** Phenotypes of transplastomic tobacco lines grown in sterile culture on sucrose-containing medium. Typical phenotypes are shown. At least two transplastomic lines were generated per construct. Scale bar: 1 cm. **(C)** Light–response curves of linear electron flux (left panel) and chlorophyll-*a* fluorescence parameter qL (a measure for the redox state of the PSII acceptor side; right panel) in the wild type (*Nt*-WT) and transplastomic tobacco lines. Ten wild-type plants and 3–10 plants per transplastomic line were measured. For each construct, at least two independently generated transplastomic lines were analyzed. Average data are shown, error bars represent standard deviation. **(D)** ATP synthase activity per thylakoid membrane determined from dark-interval decay kinetics of the proton motive force-related electrochromic shift signal. Ten wild-type plants and 3–10 plants per transplastomic line were measured. For each construct, at least two independent transplastomic lines were analyzed. Columns display average data and standard deviation. Columns bearing the same letter indicate samples that were not significantly different according to one-way ANOVA with Holm–Sidak post-hoc testing. (*P* < 0.05; Supplemental Data Set 1). n.d., not determined. **(E)** Analysis of the targeted AtpB peptide by LC–MS/MS (cf. Figure 3D).

**Figure 6:**
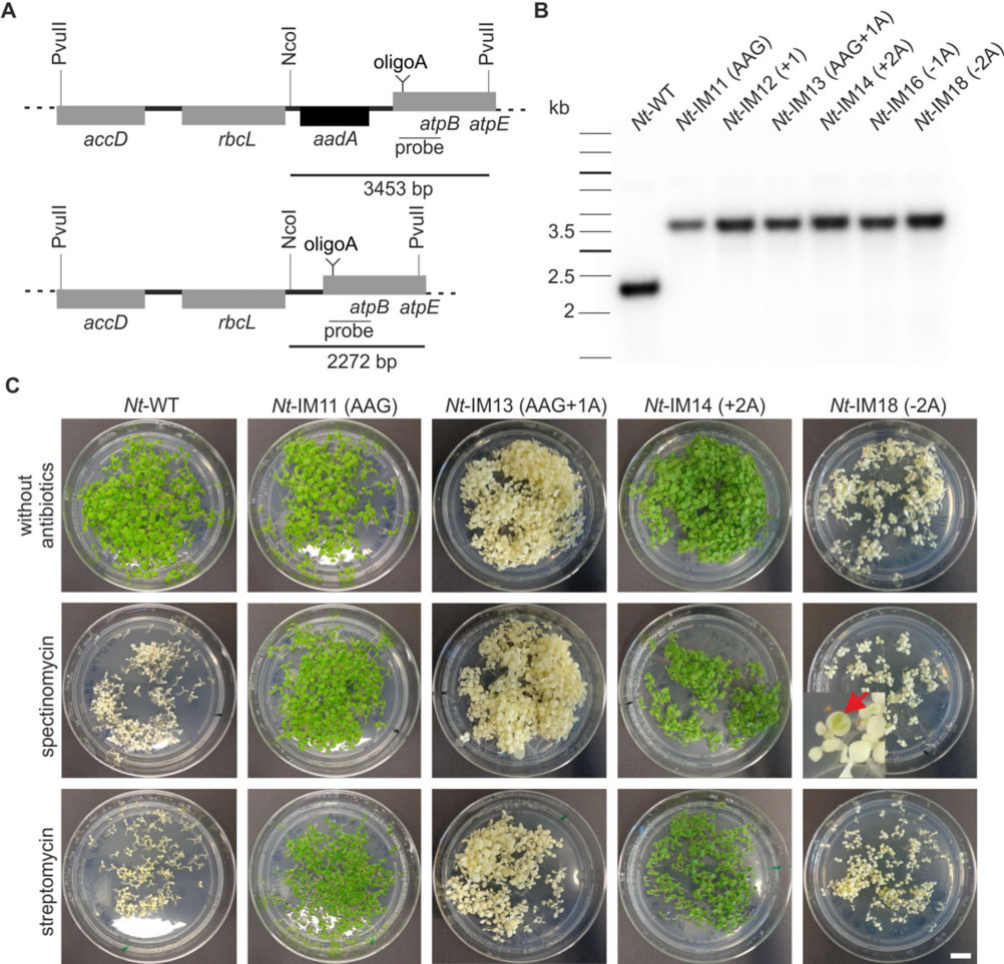
Confirmation of the homoplasmic state of transplastomic tobacco mutants. **(A)** Physical maps of the plastome region of transplastomic lines (top) and of the wild type (bottom) (modified after Rott et al., 2011). Tobacco genes are shown as grey boxes, the black box represents the *aadA* cassette. Restriction sites used for restriction fragment length polymorphism (RFLP) analysis are indicated. **(B)** RFLP analysis of transplastomic plants. DNA was digested with PvuII and NcoI. The location of the hybridization probe is indicated in (A). The wild type (*Nt*-WT) shows the expected restriction fragment of 2.3 kb, while all transformants show only the transplastomic fragment of 3.5 kb, confirming their homoplasmic state. **(C)** Inheritance test. Seeds were germinated on synthetic medium without antibiotic (control) or medium supplemented with either streptomycin or spectinomycin. *Nt*-IM11 (AAG) and *Nt*-IM14 (+2A) seeds were derived from homoplasmic plants, *Nt*-IM13 (AAG+1A) and *Nt*-IM18 (−2A) seeds were obtained from periclinal chimeras with mutated chloroplasts in their L2 layer (cf. Greiner, 2012 and “Materials and methods” section). The red arrow indicates rare green spots on *Nt*-IM18 (−2A) mutant leaves. Scale bar: 1 cm.

Replacement of three adenines with guanines in third codon position resulted in a wild-type-like phenotype [*Nt*-IM11 (AAG); Figure 5B], as expected. In contrast, *Nt-*IM12 (+1A) plants carrying the same mutation as I-iota (+1A) resembled the mottled phenotype of the *Oenothera* mutant (Figures 1A, 1C and 5B). Similar to *Oenothera* I-iota plants, transplastomic *Nt*-IM12 (+1A) plants were unable to grow autotrophically. Interestingly, combined disruption of the oligoA stretch and insertion of the I-iota mutation led to an albino phenotype. Transplastomic *Nt*-IM13 (AAG+1A) plants were white and resembled the albino phenotypes of transplastomic *ΔatpB* knock-out mutants (Hager, 2002) and other loss-of-function mutants of the chloroplast ATP synthase (Karcher and Bock, 2002; Schmitz-Linneweber et al., 2005). This observation indicates that the oligoA stretch is of crucial importance for the frame-correcting mechanism.

Next, we analyzed photosynthetic parameters in the transplastomic tobacco plants to assess their physiological similarity to the *Oenothera* I-iota mutant. No significant differences in linear electron flux and the redox state of the PSII acceptor side (qL) were detected between the wild type (*Nt*-WT), the *aadA-atpB*, and the *Nt*-IM11 lines (Figure 5C). Interestingly, although chlorophyll content was similar in *Nt*-IM12 (+1A; the I-iota mutation) and the control lines (Table 3), all other parameters were reduced in *Nt*-IM12 (+1A). Linear electron flux was strongly decreased in *Nt-*IM12 (+1A), and the PSII acceptor side became reduced already in very low actinic light intensities (Figure 5C), similar to the *Oenothera* I-iota mutant. Also, F_V_/F_M_ was strongly reduced in *Nt*-IM12 (+1A), again pointing to PSII photoinhibition and the presence of uncoupled antenna proteins. This conclusion is further supported by the strongly decreased chlorophyll *a/b* ratio of *Nt*-IM12 (+1A), arguing for a predominant loss of the photosystems (Table 3). No analysis of photosynthetic performance was possible for *Nt*-IM13 (AAG+1A), since the plants were completely white. In addition, activity of the ATP synthase was determined. As expected, no significant differences in ATP synthase activity between the wild type, the *aadA-atpB* control plants and *Nt*-IM11 (AAG) plants were detected (Figure 5D). In contrast, the line *Nt*-IM12 (+1A) displayed a strongly reduced activity of ATP synthase.

The observed phenotypes of the transplastomic tobacco lines suggested a varying degree of AtpB correction in the different lines. To explore the correlation between phenotype of the plants and synthesis of full-length AtpB protein, the accumulation of AtpB protein was analyzed by tryptic in-gel digestion followed by LC–MS/MS analysis. Full-length AtpB was detected in the wild type (*Nt*-WT), the control line *aadA-atpB*, and the *Nt*-IM11 (AAG) and *Nt*-IM12 (+1A) mutants. No AtpB was detected in the white *Nt*-IM13 (AAG+1A) line (Table 2).

To compare the mechanism of reading frame correction with the I-iota mutant, the protein bands corresponding to AtpB were subjected to Glu-C digestion and analyzed by LC–MS/MS. As in I-iota, we identified identical peptides covering the slippery site in *Nt*-IM12 (+1A) and wild-type tissue (Figure 5E;Supplemental Figure 3), suggesting operation of +1 correction also in the chloroplasts of tobacco (Table 1). No peptides derived from other reading frames could be detected (Table 1; Supplemental Table 1).

### Influence of mutations in the oligoA stretch on frameshift efficiency in *E. coli*

The phenotype of the *Oenothera* I-iota mutant and the transplastomic tobacco *Nt*-IM12 (+1A) line strongly suggests the presence of +1 frame correction in the chloroplasts of seed plants. Chloroplasts are of prokaryotic origin (Bock and Timmis, 2008) and have a bacterial-like translation machinery (Zoschke and Bock, 2018). Moreover, structure and subunit composition of CF_1_F_0_ resembles the ATP synthases of bacteria (Hahn et al., 2018). To test the possibility that the *atpB* reading frame correction also occurs in bacteria and, if it does, precisely measure its efficiency, a dual-luciferase reporter system in *E. coli* was employed (Kramer and Farabaugh, 2007). The dual-luciferase reporter assay is a powerful tool to analyze frameshifting efficiencies in various organisms and systems (Grentzmann et al., 1998; Kramer and Farabaugh, 2007; Ketteler, 2012). The assay is based on the Renilla luciferase serving as expression control, whereas the activity of the firefly luciferase depends on the expression of the test sequence (Grentzmann et al., 1998; Kramer and Farabaugh, 2007). Thus, the activity of the firefly luciferase relative to the Renilla luciferase reflects the translation efficiency (Grentzmann et al., 1998).

To determine the translation efficiency of our various *atpB* sequences, 150 nt of the 5′-part of the *atpB* gene from *Oenothera* and tobacco were cloned into the pEK4 plasmid (Kramer and Farabaugh, 2007) between the Renilla and firefly luciferase genes. In addition, several constructs varying in the length of the oligoA stretch or harboring a disrupted oligoA stretch (by placing guanines into the third codon position; see above) were generated (Supplemental Table 2). The translation efficiency measured in the constructs containing the *Oenothera* or tobacco wild-type sequence (pEK4-*Nt*-WT and pEK4-*Oe*-WT) was set as 100%. The pEK4-*Nt*-IM11 (AAG) construct, where the oligoA track of the tobacco *Nt*-WT sequence is destroyed by guanines, displayed an even enhanced efficiency compared to the wild-type sequences (Figure 7). In contrast, single adenine insertions caused a decrease in firefly luciferase activity relative to Renilla luciferase in *Oenothera* and the analogous tobacco constructs [pEK4-*Oe*-WT versus pEK4-I-iota (+1) and pEK4-*Nt*-WT versus pEK4-*Nt*-IM12 (+1A); Figure 7]. Similar to the *in planta* situation, disruption of the oligoA stretch in the single adenine insertion construct resulted in a significant reduction of the frameshift efficiency [pEK4-*Nt*-IM12 (+1A) versus pEK4-*Nt*-IM13 (AAG+1A)]. Interestingly, +2A insertion in the oligoA stretch [pEK4-*Nt*-IM14 (+2A)] improved the translation efficiency by ca. 40%. Single or double adenine deletions [pEK4-*Nt*-IM16 (−1A) and pEK4-*Nt*-IM18 (−2A), respectively] did not exhibit effective frame correction and showed only approximately 5% of residual activity (Figure 7). In conclusion, also in the *E. coli*-based assay system, frame correction was detected in all constructs containing the single adenine insertion. Surprisingly, an even higher translation efficiency was detected in pEK4-*Nt*-IM14 construct with two adenines inserted.

**Figure 7:**
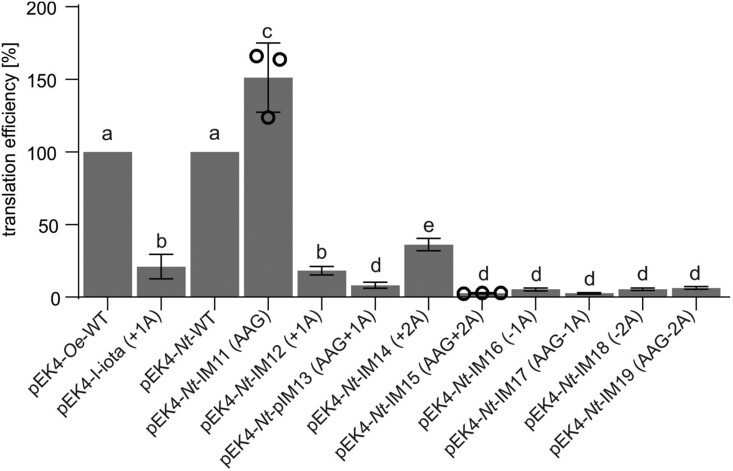
Impact of frameshift mutations in the oligoA stretch on translation efficiency as measured with the dual-luciferase system in *E. coli*. Three bacterial colonies were analyzed for pEK4-*Nt*-IM11 and pEK4-*Nt*-IM15 (Data points are shown) and 6–9 colonies were tested for other constructs. Columns display average data and standard deviation. Columns bearing the same letter indicate samples that were not significantly different according to one-way ANOVA with the Holm–Sidak post hoc testing (*P* < 0.05; Supplemental Data Set 1).

### Transplastomic tobacco plants with varying lengths of the oligoA stretch

The effective frame correction observed with the pEK4-*Nt*-IM14 (+2A) construct (Figure 7) raised the question how a+2A insertion into the oligoA stretch of *atpB* would affect the plant phenotype. To address this question, additional transplastomic tobacco lines were designed. Line *Nt*-IM14 (+2A) carries a+2A mutation, while lines *Nt*-IM16 (−1A) and *Nt*-IM18 (−2A) carry deletions of one or two adenines, respectively (Figure 8A). Homoplasmy of the generated transplastomic mutants was tested by restriction fragment length polymorphism analysis (Figure 6B), and presence of the mutations was verified by Sanger sequencing. Interestingly, *Nt-*IM14 (+2A) plants were indistinguishable from the wild type under mixotrophic conditions (Figure 8B). Moreover, although clearly retarded in growth, they were even able to grow photoautotrophically (Figure 9A). Compared to the wild type, *Nt*-IM14 (+2A) plants flowered 5–8 months later but produced viable seeds that could be used for seed tests for homoplasmy (Figure 6C; cf. “Materials and methods” section). Therefore, for *Nt*-IM14 (+2A) and the control lines *aadA*-*atpB* and *Nt*-IM11 (AAG) the full set of physiological parameters could be determined under both mixotrophic and autotrophic conditions (Figures 8C and 9C). Interestingly, plants with a single adenine deletion [*Nt*-IM16 (−1A)] displayed a similar phenotype as plants with a single adenine insertion [*Nt*-IM16 (−1A) versus *Nt*-IM12 (+1A), Figures 8B versus 5B]. Both mutants exhibited pale green leaves with white areas. In contrast, the *Nt*-IM18 (−2A) line with the 2A deletion did not resemble the *Nt*-IM14 (+2A) phenotype. *Nt*-IM18 (−2A) plants were white with small green spots (Figure 8B), suggesting that the double adenine deletion cannot be efficiently compensated by recoding. Similar to *Nt*-IM13 (AAG+1A), homoplasmic seeds could be obtained from stable periclinal chimeras (cf. “Materials and methods” section). Seedlings that germinated in the presence or absence of spectinomycin were white with occasional green spots (Figure 6C). The visually observed phenotypes were further confirmed by the analysis of photosynthetic parameters: *Nt*-IM14 (+2) plants displayed a higher chlorophyll content compared to the other plant lines (Table 3), and the photosynthetic parameters of *Nt*-IM12 (+1A) and *Nt*-IM16 (−1A) were similar (Table 3 and Figures 8C versus 5C), suggesting comparable *atpB* correction efficiency. *Nt*-IM14 (+2A) displayed intermediate photosynthetic behavior when compared to *Nt*-WT and *Nt*-IM12 (+1A) or *Nt*-IM16 (−1A) plants (Figures 8C, 9C and Table 3).

**Figure 8:**
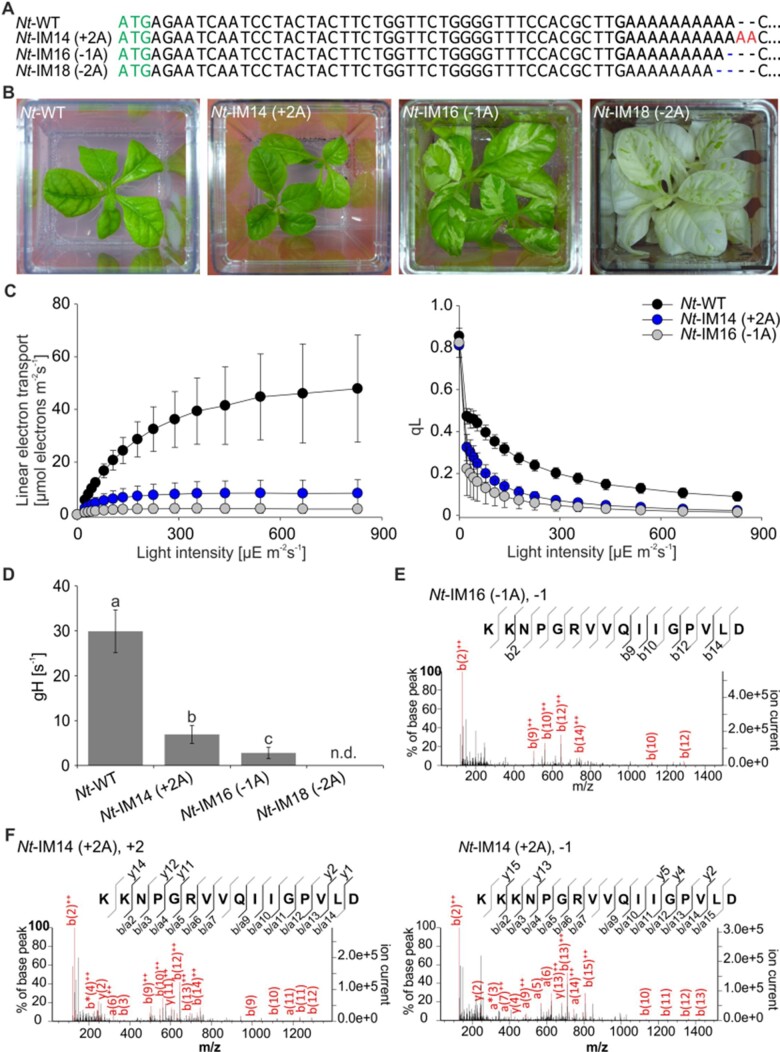
Generation and characterization of transplastomic tobacco mutants varying in the length of the oligoA stretch in *atpB*. **(A)** Mutants generated to analyze recoding in tobacco. Red letters represent insertions, blue dashes denote deletions. **(B)** Phenotypes of transplastomic tobacco lines grown in sterile culture. Typical phenotypes are shown. At least two transplastomic lines were generated per construct. Scale bar: 1 cm. **(C)** Light–response curves of linear electron flux (left panel) and the chlorophyll *a* fluorescence parameter qL (a measure for the redox state of the PSII acceptor side; right panel) in the wild type (*Nt*-WT) and transplastomic tobacco lines. Ten wild-type plants and 3–10 plants per transplastomic line were measured. For each construct, at least two independently generated transplastomic lines were analyzed. Average data are shown, error bars represent standard deviation. **(D)** ATP synthase activity per thylakoid membrane determined from dark-interval decay kinetics of the proton motive force-related electrochromic shift signal. Ten wild-type plants and 3–5 plants per transplastomic line were measured. For each construct, at least two independent transplastomic lines were analyzed. Columns display average data and standard deviation. Columns bearing the same letter indicate samples that were not significantly different according to one-way ANOVA with the Holm–Sidak post hoc testing (*P* < 0.05, Supplemental Data Set 1). n.d., not determined. **(E, F)** Analysis of peptides derived from the slippery site in AtpB by LC–MS/MS (for detail see Figure 3D). −1 and +2 denote the direction of translational recoding (for detail see “Introduction” section).

**Figure 9:**
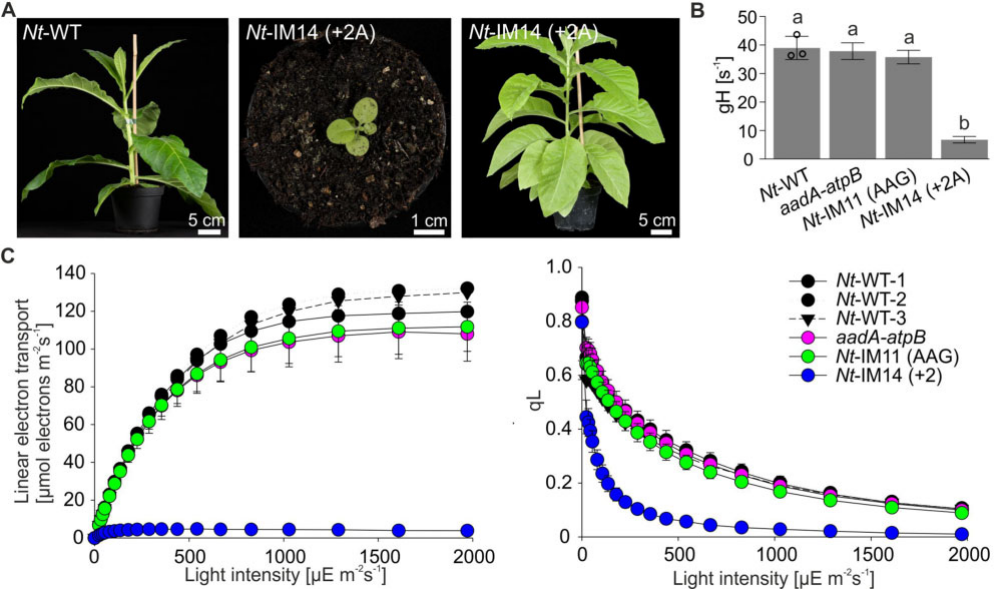
Correction of a+2 insertion in the *atpB* gene by recoding enables photoautotrophic growth. **(A)** Growth phenotypes of wild-type (*Nt-*WT) and *Nt*-IM14 (+2A) plants cultivated in the greenhouse. Pictures were taken 7 weeks (left and middle) and 3 months (right photo) after germination. Note the strong retardation in growth of *Nt*-IM14 (+2A) mutants compared to the wild type. Typical phenotypes are shown. Three transplastomic lines of *Nt*-IM14 were grown photoauthotrophicaly showing similar phenotypes. **(B)** ATP synthase activity. *Nt*-WT, *aadA-atpB*, and *Nt*-IM11 (AAG) plants were 7 weeks old, *Nt*-IM14 (+2A) plants were 14 weeks old. Three wild-type plants (data points are shown) and 3–5 plants per transplastomic line were measured. For each construct, at least two independent transplastomic lines were analyzed. Columns display average data and standard deviation. Columns bearing the same letter indicate samples that were not significantly different according to one-way ANOVA with Holm–Sidak post hoc testing (*P* < 0.05; Supplemental Data Set 1). **(C)** Light–response curves of linear electron flux (left panel) and the chlorophyll *a* fluorescence parameter qL (a measure for the redox state of the PSII acceptor side; right panel) in the wild type and transplastomic tobacco lines. Three wild-type plants (data points are shown) and 3–5 plants per transplastomic line were measured. For each construct, at least two independently generated transplastomic lines were analyzed. Average data are shown, error bars represent standard deviation.

Next, ATP synthase activity was assessed in the new set of transplastomic lines. The green *Nt*-IM14 (+2A) line displayed the highest ATP synthase activity among all mutants containing frameshift mutations (Figures 8D and 5D). In fact, when compared under mixotrophic and autotrophic growth conditions, *Nt*-IM14 (+2A) plants showed similar ATP synthase activity (19% and 17%, respectively; Figures 8D and 9B). In contrast, ATP synthase activity in *Nt*-IM16 (−1A) was at the detection limit, close to the decay kinetics of thylakoids with inactive ATP synthase. The ECS was not measurable in white *Nt*-IM18 (−2A) plants.

Finally, proteomic analysis of the transplastomic mutants was performed by tryptic in-gel digestion followed by LC–MS/MS. The AtpB protein was detected in all newly generated lines (Table 2). Peptides derived from the mutated site were generated by Glu-C digestion to gain insight into the correction mechanisms operating (Table 1). Peptides clarifying the amino acid sequence specified by the *atpB* mutations introduced into the transplastomic lines were identified in *Nt*-IM16 (−1A) and *Nt*-IM14 (+2A) (Figure 8E and 8F). Interestingly, both +2 and −1 corrections were detected in the Nt-IM14 (+2A) line, as evidenced by identification of peptides with two or three lysines (Figure 8F). A peptide resulting from −1 correction resulted in two lysine stretch was detected in *Nt*-IM16 (−1A) (Figure 8E and Table 1). No targeted peptides were detected in the white line *Nt*-IM18 (−2A).

### Analysis of the *atpB* cDNA in *Oenothera* and transplastomic tobacco plants

Since RNA polymerase stuttering and ribosomal frameshifting can both occur at homopolymeric sequence motifs, it can be challenging to unambiguously distinguish between the two recoding mechanisms (Atkins et al., 2016). Transcriptional slippage generates a heterogeneous population of mRNAs that can be detected by cDNA analysis. Although seemingly straightforward, the high error rate exhibited by reverse transcriptases can complicate this approach (Roberts et al., 1988). To minimize this problem, we used a high-fidelity reverse transcriptase (a variant of Moloney murine leukemia virus reverse transcriptase, MMLV-RT, that was combined with a 3′–5′ exonuclease domain for proofreading). The enzyme exhibits an error rate that is three times lower than that of conventional reverse transcriptases (Arezi and Hogrefe, 2007). cDNA sequence analyses revealed that, in all transplastomic lines, the mutations introduced into the *atpB* locus were faithfully represented in the mature *atpB* mRNAs (Figure 10A). No sequence shifts or double peaks were detected in any of the chromatograms. A similar situation was observed for I-iota and the corresponding *Oenothera* wild type (*Oe*-WT), although an overall somewhat more noisy sequence was obtained here for both the wild type and the mutant (Figure 10B). Furthermore, no transcriptional slippage was detected in I-iota tissues from different developmental stages (Supplemental Figure 4). To estimate the accuracy of cDNA synthesis and the sensitivity of the assay, we mimicked RNA polymerase stuttering by mixing total RNA from *Nt*-WT and *Nt*-IM14 (+2A) in different ratios followed by reverse transcription and sequence analysis of the amplified cDNA population (Figure 10C). When the amount of added *Nt*-WT RNA was ≥5%, an additional peak in the sequence chromatogram appeared (Figure 10C), revealing the presence of the heterogeneous *atpB* mRNA population. Absence of the double peak from the *atpB* sequences of the *Oenothera* I-iota mutant and the analogous transplastomic tobacco lines indicates that the level of RNA polymerase stuttering, if occurring at all, is below the 5% detection threshold (Supplemental Figure 5). Given that the ATP synthase activity in the mutants reaches levels of more than 17% [in *Nt*-IM14 (+2A); Figures 8D and 9B], frameshift correction must occur mostly, if not exclusively, by ribosome frameshifting.

**Figure 10:**
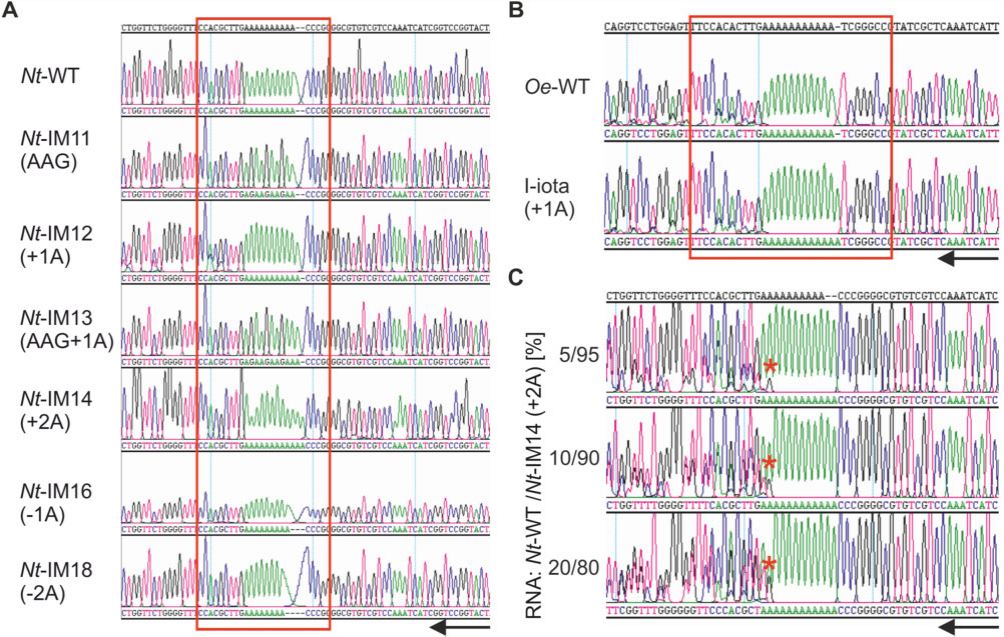
cDNA analysis to test for RNA polymerase stuttering in *atpB*. **(A)** cDNA analysis of transplastomic tobacco plants and the wild type (*Nt*-WT). The amplified cDNA population was sequenced, and typical Sanger sequencing chromatograms are presented. The sequence containing the oligoA stretch is boxed in red. **(B)** cDNA analysis of *Oenothera* wild-type (*Oe*-WT) and I-iota (+1) plants. **(C)** Sensitivity test for the detection of RNA polymerase stuttering. RNA was extracted from the wild type (*Nt*-WT) and the *Nt*-IM14 (+2A) transplastomic line, and the RNA samples were mixed in different ratios (indicated in %). cDNA was synthesized, amplified, and sequenced. Red asterisks indicate the additional peak, corresponding to the spiked-in wild-type RNA. Black arrows indicate the direction of the sequencing reaction. A–C, Typical chromatograms are shown. cDNA analyses were repeated at least once with separate batches of plants.

## Discussion

Plastome mutants are an important tool for the analysis of non-Mendelian inheritance, the study of photosynthesis, and the analysis of the mechanisms of chloroplast gene expression (Greiner, 2012; Sobanski et al., 2019). Frameshift mutations usually lead to disruption of protein function, unless they are transcriptionally or translationally corrected (Ketteler, 2012; Atkins et al., 2016). Although such recoding is believed to exist in almost all organisms, only very few examples have been found in plants to date (see “Introduction” section).

### Efficient recoding requires a homopolymeric sequence

Recoding based on transcriptional slippage relies on homopolymeric sequences (Baranov et al., 2005), while PRF can occur on either homopolymeric or heteropolymeric slip sites (Baranov et al., 2011). Transplastomic tobacco plants, where in addition to the frameshift mutation, adenines at the third codon position of the oligoA stretch had been replaced by guanines, resulted in white plants [*Nt*-IM13 (AAG+1A); Figure 5B], suggesting complete loss of ATP synthase (Hager, 2002; Schmitz-Linneweber et al., 2005). Strongly reduced translation efficiency of these constructs was also observed with the dual-luciferase assay in *E. coli* (Figure 7). These findings indicate that frameshift compensation in *atpB* requires a homopolymeric tract. It was shown in bacteria that mRNAs encoding iterated lysine codons (either AAA or AAG) differ remarkably in efficiency and accuracy of protein synthesis. The presence of repetitive AAA codons reduced protein expression compared to the presence of synonymous AAG codons. This is because 70S ribosomes read AAA and AAG codons differently. Ribosomes that encounter multiple AAA codons undergo sliding, whereas no sliding occurs on AAG codons (Koutmou et al., 2015). These findings are in line with our observation that, in the dual-luciferase assay, an enhanced translation efficiency was detected in pEK4-*Nt*-IM11 (AAG) compared to the wild-type sequence that carries the oligoA stretch [pEK4-*Nt*-WT] (Figure 7).

Interestingly, the −2A deletion [*Nt*-IM18 (−2A)] results in an albino phenotype with rare green spots (Figures 8B and 6C). *Nt*-IM18 (−2A) plants are phenotypically more similar to *Nt*-IM13 (AAG+1A), and no AtpB peptides were detected in *Nt*-IM18 (−2A). This indicates that −2 or +1 ribosomal frameshifting are not active in *Nt*-IM18 (−2A) (Table 1), and an A_8_ homopolymeric tract is also insufficient to effectively induce transcriptional slippage. This is not entirely unexpected in that the propensity of RNA polymerase to stutter is known to be strongly correlated with the length of the (homopolymeric) slippery site (Lin et al., 2015).

Furthermore, the phenotypes of I-iota and tobacco transplastomic lines represent a dose-threshold dependency, previously claimed, for example, for the *immutans* mutant of *Arabidopsis* with green and white leaf sectors (Wetzel et al., 1994; Pogorelko et al., 2016). According to our data, accumulation of a certain amount of ATP synthase seems to be necessary for greening, which is in line with a developmental gradient of thylakoid membrane formation (Figures 2C to 2F) in dependency on the white/green phenotype (Figure 1A). This places assembly of the ATP synthase very early in thylakoid formation, if it is not even the initial step. Also, in etioplasts, ATP synthase (and the cytochrome *b_6_f* complex) are the first complexes to fully assemble, while the photosystems only accumulate in the light (Kanervo et al., 2008; Reisinger et al., 2008; Plöscher et al., 2011; Armarego-Marriott et al., 2019).

### What mechanism of recoding is acting in I-iota and transplastomic tobacco lines?

It can be challenging to distinguish between transcriptional slippage and PRF when monitoring it at a low level (Atkins et al., 2016). PRF often results in the formation of more than one protein. Likely due to instability of the truncated AtpB protein (Nishimura et al., 2017), our proteomic analyses detected only peptides corresponding to the corrected reading frame. However, two corrected protein variants (−1 and +2) were found for *Nt-*IM14 (+2A) (Figure 8F), lending circumstantial support to the operation of ribosomal frameshifting rather than RNA polymerase stuttering. Analysis of the predicted mRNA secondary structure revealed the presence of a stem–loop-type structure downstream the oligoA stretch that can serve as slip site for the ribosome (Figures 3C and 4).

We have assessed the possibility of transcriptional slippage by cDNA analysis of the various mutants. No indication of transcriptional slippage for *Nt*-IM12 (+1A) and I-iota (+1A) (Figures 10A and 10B) was obtained, but spike-in experiments mixing of *Nt*-IM12 (+1A) or I-iota (+1A) with the corresponding wild-type RNAs revealed a detection limit of approximately 15% (Supplemental Figure 5). Since the frame correction in *Nt*-IM12 (+1A) is estimated to be below 10% (Figure 5D), the operation of transcriptional slippage cannot be definitively excluded for *Nt*-IM12 (+1A).

Strikingly, in the transplastomic tobacco mutants, the +2A insertion into the oligoA tract is more efficiently corrected than the +1A insertion [*Nt*-IM14 (+2A) versus *Nt*-IM12 (+1A), Figures 8C and 5C]. *Nt*-IM14 (+2A) plants are phenotypically most similar to the wild type under mixotrophic conditions, and also can grow photoautotrophically and produce seeds (Figures 8B and 9A). Regarding their photosynthetic parameters, *Nt*-IM14 (+2A) plants display an intermediate phenotype between the wild type and *Nt*-IM12 (+1A) plants (Figures 8C, 9C and Table 3). In *Nt*-IM14 (+2A) plants, AtpB peptides with two or three lysines were detected, suggesting two modes of correction: +2 and −1 (Figure 8F). In other systems, several cases have been reported where two different ribosomal frameshifts can occur at the same site (Caliskan et al., 2017; Li et al., 2019). As for *Nt*-IM12 (+1A), cDNA analysis revealed no indication of transcriptional slippage in *Nt*-IM14 (+2A) plants (Figure 10A). Importantly, in the cDNA sensitivity test, 5% of added RNA was detectable, when wild-type and *Nt*-IM14 (+2A) RNAs were mixed (Figure 10C). Together with the absence of detectable RNA polymerase stuttering, this high sensitivity suggests that the correction occurs at the level of translation (i.e. by PRF). Work in microbial systems has revealed that the efficiency of alternative decoding can be highly variable, ranging from a few percent to up to 80% (Baranov et al., 2005; Caliskan et al., 2015). The frameshifting efficiency in *Nt*-IM14 (+2A) measured by the dual-luciferase reporter assay in *E. coli* was approximately 40% (Figure 7). The efficiency *in planta* may be lower, as evidenced by the measured ATP synthase activity in *Nt*-IM14 (+2A) plants, which was 18% of the activity in wild-type plants (Figures 8D and 9B).

### Functional significance of recoding in chloroplasts

In plastid genomes, homopolymeric nucleotide stretches are hotspots of mutations (Massouh et al., 2016). Homopolymeric tracts are selected against in bacterial genomes, but are retained more frequently in the genomes of endosymbionts. Transcriptional slippage has been proposed to play a critical role in rescuing gene function (Tamas et al., 2008; Wernegreen et al., 2010). Here polymerase stuttering at homopolymeric tracts can be considered as a mutation-compensating mechanism (Tamas et al., 2008). Whether or not transcriptional slippage occurs in chloroplasts and compensates frameshift mutations and/or generates additional transcript diversity, is currently unclear. If it occurs at the oligoA stretch near to 5′-end of *atpB*, its frequency is too low to be detected with the sensitivity of cDNA analysis (Figure 10;Supplemental Figure 5).

PRF results in the formation of additional protein products but can also correct indels at the level of translation (Ketteler, 2012; Atkins et al., 2016). Usually, ribosomal frameshifting is tightly regulated. Specific metabolites and aminoacyl-tRNA supply can affect the efficiency of ribosomal frameshifting (Ivanov et al., 2000; Caliskan et al., 2017). When we searched for other possible examples of alternative genetic decoding in the chloroplast, a sequence motif in the *cemA* (*ycf10*) gene (coding for an inner envelope protein likely involved in CO_2_ uptake; Rolland et al., 1997) was identified. In contrast to *O. elata* (which was investigated here), *O. glazioviana* and *O. parviflora* (*Oenothera* subgenus *Oenothera*), as well as *O. villaricae* and *O. picensis* (*Oenothera* subgenus *Munzia*) contain two ATG codons embedded in an oligoA stretch within the 5′ region of *cemA*. The oligoA stretches of the two pairs of species differ by an indel of 3 bp, and only the second ATG is in frame. To be used, the first ATG would require a −1 or +2 frameshift. Similar to *atpB*, the oligoA stretch could serve as slippery sequence motif for recoding (Greiner et al., 2008). To what extent recoding can result under certain experimental conditions even in gene fusion products, as suggested by the AtpB/AtpE fusion observed by Sears and Herrmann (1985) (also see “Results” section), remains a matter of speculation.

Previous reports on the possibility of recoding in plants were based on bioinformatic prediction (Lin et al., 2015; van der Horst et al., 2019), *in vitro* analysis (Napthine et al., 2003) or indirect genetic evidence (Kohl and Bock, 2009). The data presented in this study provide clear genetic and biochemical evidence for the presence of recoding in the chloroplast *atpB* gene both in the spontaneous plastome mutant I-iota of *Oenothera* and a set of transplastomic tobacco plants. Its presence in chloroplasts may be a relic from the bacterial past of the chloroplast (Lin et al., 2015). To what extent the compensation for mutations by frameshifting is dependent upon the environmental conditions and/or the physiological status of the chloroplast, is an interesting question to be addressed in future studies.

## Materials and methods

### Plant material and growth conditions

*Oenothera* (evening primrose) wild type (*Oe*-WT = *O. elata* ssp. *hookeri* strain johansen Standard equipped with the chloroplast genome of *O. elata* ssp. *hookeri* strain hookeri de Vries = johansen Standard I-hookdV) and the I-iota mutant (mutation in chloroplast genome of johansen Standard I-hookdV) were previously described in Massouh et al. (2016) and cultivated according to Greiner and Köhl (2014). In *Oenothera*, photosynthetically incompetent chloroplast mutants can be kept as variegated plants, containing a mixed population of green nursery chloroplasts and mutated plastids. These plants are viable in soil because the green tissue feeds the nonphotosynthetic mutant leaf tissue (Figure 1A). The combination of two chloroplast types in one plant is achieved by taking advantage of biparental chloroplast transmission, which is the rule in evening primroses. To this end, a maintainer strain containing green nursery plastids is crossed to the chloroplast mutant. This results in variegated offspring because vegetative sorting (sorting-out) of the two chloroplast types leads to tissues homoplasmic for either green or mutant chloroplasts in the same plant. Biparental transmission of plastids in *Oenothera* displays, however, maternal dominance. Consequently, the F1 generation offspring is usually either homoplasmic for the maternal plastome or heteroplasmic for the paternal and maternal plastomes. To increase the frequency of variegated (heteroplasmic) offspring in F1, a johansen Standard line was equipped with the slowly multiplying plastome IV of *O. parviflora* strain atrovirens Standard (IV-atroSt) as green nursery plastome (cf. Sobanski et al., 2019). This line was then used as seed parent in crosses with the faster multiplying I-iota plastome. Resulting F1 generations display up to 100% variegation for green and mutated plastomes in the progeny. Homoplasmic mutant tissue generated this way was used for molecular and physiological analyses in this work. For details on the genetics, see (Kutzelnigg and Stubbe, 1974; Kirk and Telney-Basset, 1978; Stubbe and Herrmann, 1982; Greiner, 2012; Sobanski et al., 2019).

For plant growth, *Oenothera* seeds were soaked in water for 12–16 h at 4°C, germinated on wet filter paper at 27°C in the light (50 µE m^−2^ s^−1^, OSRAM L36W/840 LUMILUX Cool White), followed by seedling transfer to soil (details in Greiner and Köhl, 2014). About 1 month after germination (early rosette stage) plants were vernalized at 4°C for 7 days under a 10 h light (100–150 µE m^−2^ s^−1^, Valoya, AP673L)/14 h dark regime. Plants were then returned to the greenhouse and cultivated under long-day conditions (16 h light, 180–220 µE m^−2^ s^−1^ [Philips, SON-T-PIA AGRO 400 W], 22°C /8-h dark, 20°C, humidity 50%).

Homoplasmic I-iota plants and wild-type control plants were cultivated in Magenta vessels (Sigma-Aldrich) under aseptic conditions (sterile culture). Seeds were sterilized following a vapor-phase sterilization protocol. Seeds in open microcentrifuge tubes were placed in a desiccator jar together with a glass beaker containing 100 mL of 13% [v/v] NaClO. Prior to sealing the desiccator jar, 3 mL concentrated HCl was carefully added to the bleach. Seeds were then sterilized in developing chlorine fumes for 5 h, followed by seeds ventilation in open tubes in a laminar flow hood for several hours to completely eliminate the chlorine gas. Subsequently, seeds were plated on germination medium containing ½ MS salts (Murashige and Skoog, 1962), 0.5% [w/v] Agargel (Sigma Aldrich), and 0.05% [v/v] Plant Preservative Mixture (Plant Cell Technology), adjusted to pH 5.8 with KOH, and incubated for 27°C in the light (50 µE m^−2^ s^−1^, OSRAM L36W/840 LUMILUX Cool White). After 1 week, seedlings were transferred to ½ MS medium with Agargel (see above) supplemented with 1.75% [w/v] glucose and cultivated at 16 h light (50 µE m^−2^ s^−1^, OSRAM L36W/840 LUMILUX Cool White)/8 h dark and 24°C.

*Nicotiana tabacum* cv. Petit Havana (*Nt*-WT) and *atpB*-*aadA* control plants (Rott et al., 2011) were raised from seeds in soil under controlled growth conditions (16 h light, 150–180 µE m^−2^ s^−1^ [Philips, SON-T-PIA AGRO 400W], 25°C/8 h dark, 20°C; humidity 60%). Young plants were transferred to the small pots (6 cm diameter) on “Einheitserde Typ P”-soil (Einheitserde, Germany) and watered once with water supplemented with Previcur (15 mL in 10 L, Bayer, Germany). Later plants were watered manually once per day. After ca. 4 weeks plants were transferred to the bigger pots (13 cm diameter) filled with mixture of "Einheitserde Typ T"-soil (Germany)/vermiculite in the ratio 1:3. Mature plants were watered automatically via a drip system (Dosatron, Germany) containing fertilizer (0.7 g L^−1^ Hakaphos rot, COMPO GmbH, Germany). Transplastomic tobacco lines were cultivated in Magenta boxes on agar-solidified MS medium containing 3% [w/v] sucrose and supplemented with 500 mg·L^−1^ spectinomycin under 16-h light (50 µmol m^−2^s^−1^, OSRAM L36W/840 LUMILUX Cool White)/8-h dark, 24°C. For the inheritance tests (Svab and Maliga, 1993; Bock, 2001), seeds were surface-sterilized by soaking in 70% [v/v] ethanol for two minutes, followed by soaking in 3% [v/v] NaClO containing a drop of Triton X-100 for 15 min. The seeds were then rinsed with sterilized water 5–7 times and plated on MS-based selective media containing 500 mg·L^-1^ spectinomycin, or 500 mg·L^−1^ streptomycin (Rott et al., 2011). Homoplasmic *Nt*-IM13 (AAG+1A) and *Nt*-IM18 (−2A) seeds were obtained from stable *Nt*-IM13 (AAG+1A)/*Nt*-WT and *Nt*-IM18 (−2A)/*Nt*-WT periclinal chimeras (plants with a white leaf border carrying mutated chloroplasts and a green leaf blade with wild-type chloroplasts; cf. Greiner 2012), respectively.

### Test for homoplasmic I-iota tissue

Homoplasy of leaf sectors of I-iota was assessed using a sequence length polymorphism in the *clpP* gene that distinguishes the nursery plastome IV-atroSt (GenBank accession number EU262891.2) from the I-iota plastome that derives from I-hookdV (GenBank accession numbers KT881170.1; Massouh et al. 2016). DNA from small leaf pieces was isolated using Phire Plant Direct PCR kit (ThermoFisher Scientific). The marker was amplified by standard PCR, employing the primer pair clpP_IvsIV_for and clpP_IvsIV_rev (Supplemental Table 3; cf. Figure 1B).

### Generation of transplastomic tobacco lines

Point mutations in *atpB* were introduced by site-directed mutagenesis. 177 bp from the 5′-part of *atpB* were cloned into the pEXA vector (Eurofins) and used as a template. Primer pairs used for PCR are listed in Supplemental Table 3. Reactions were carried out employing Phusion High Fidelity DNA polymerase according to the manufacturer’s instructions (ThermoFisher Scientific). Nonmutated parental DNA was digested with the restriction enzyme DpnI (ThermoFisher Scientific) for 1 h at 37°C. DpnI-treated DNA was transformed into DH5-alpha chemically competent cells and plated on LB medium supplemented with 100 µg mL^−1^ ampicillin. Single colonies were used for inoculation of overnight culture and plasmids isolated with the help of the QIAprep Spin Miniprep Kit (Qiagen). The presence of the mutation was verified by Sanger sequencing (LGC genomics). Positive clones were digested by CpoI and BspOI (Thermo Scientific) and the released *atpB* fragment was ligated into the similarly digested *aadA-atpB* plasmid (Figure 6A; Rott et al., 2011) using T4 ligase (Invitrogen).

Transplastomic lines were generated by biolistic transformation (Rott et al., 2011). Several homoplasmic lines were identified after the first regeneration round. Homoplasmy of the lines was verified by restriction fragment length polymorphism (RFLP) analysis (Figure 6B). Point mutations were confirmed by Sanger sequencing (LGC genomics).

### DNA gel blot and RFLP analyses

Total plant DNA was isolated by a rapid cetyltrimethylammonium bromide-based miniprep method (Doyle, 1990). DNA samples were digested with PvuII and NcoI, separated in 1% [w/v] agarose gels and blotted onto Amersham Hybond-XL membranes (GE Healthcare) according to the supplier’s recommendations. Specific probes were generated by PCR amplification (Figure 6A, primers are listed in Supplemental Table 3). For hybridization, α [^32^P] dCTP-labeled probes were generated by random priming (Megaprime DNA labeling kit; GE Healthcare). Hybridization was performed in Church Buffer (1% [w/v] bovine serum albumin (BSA), 1 mM EDTA, 7% [w/v] SDS, 0.5 M Na_2_HPO4/H_3_PO_4_, pH 7.2) at 65°C overnight. Subsequently, the membranes were briefly rinsed with Wash Solution I (0.1% [w/v] SDS, 300 mM NaCl, 30 mM sodium citrate), then incubated under continuous agitation in Wash Solution I at room temperature for 20 min, and finally washed twice with Wash Solution II (0.1% [w/v] SDS, 75 mM NaCl, 7.5 mM sodium citrate) for 15 min at 65°C. For signal detection, the membranes were incubated in phosphorimager cassettes, and the hybridization signals were visualized by scanning in a Typhoon TRIO+ Variable Mode Imager (Amersham Biosciences).

### Analysis of cDNA

RNA from *Oenothera* and tobacco leaves was extracted with the Spectrum Plant Total RNA kit (Sigma Aldrich). Genomic DNA was eliminated with the Turbo DNA-free kit (Ambion). cDNA was generated with the AccuScript High Fidelity cDNA kit (Agilent) according to the supplier’s recommendation and using gene-specific primers (Supplemental Table 3). Phusion High-Fidelity DNA polymerase (ThermoFisher Scientific) was used for subsequent PCR amplification.

### Prediction of RNA secondary structure

mRNA secondary structures were predicted with the mfold web server (Zuker, 2003). Analysis of secondary structure of a set of aligned sequences was performed using the RNAalifold web server (Bernhart et al., 2008). Sequences were aligned using Clustal Omega (Sievers et al., 2011).

### Photosynthesis measurements

Light–response curves of chlorophyll-*a* fluorescence parameters were determined at 22°C using the fiberoptics version of the Dual-PAM instrument (Heinz Walz GmbH). Linear electron flux and the redox state of the PSII acceptor side (qL) (Kramer et al., 2004) were measured after 30 min of dark adaptation. Light intensity was stepwise increased from 0 to 2,000 μE m^−2^ s^−1^, with a measuring time for each light intensity of 150 s under light-limited conditions and 60 s under light-saturated conditions. After the measurement, the chlorophyll content and chlorophyll *a/b* ratio of the measured leaf segment were determined in 80% [v/v] acetone according to (Porra et al., 1989).

Proton conductivity of the thylakoid membrane (gH^+^) was used as a measure of ATP synthase activity. It was determined on intact leaves from the decay kinetics of the ECS during a short interval of darkness. The experiments were performed at 22°C. Leaves were pre-illuminated for 5 min with saturating light (1,000 μE m^−2^ s^−1^) so that photosynthesis was fully activated and ATP synthase activity was not limited by ATP consumption by the Calvin–Benson-Bassham cycle. The saturating illumination was interrupted by 15 s intervals of darkness, and the rapid first phase of the decay kinetic of the electrochromic shift was fitted with a single exponential decay function. Depending on the mutants measured, this fit was restricted to time intervals between 200 ms [for the wild type (*Nt*-WT), *aadA-atpB* and *Nt*-IM11 (AAG)] and a maximum of 800 ms after the end of the actinic illumination [in case of mutants with very low ATP synthase activity such as *Nt*-IM12 (+1A), *Nt*-IM14 (+2A), or *Nt*-IM16 (−1A)]. The reciprocal value of the time constant was used as a measure of ATP synthase activity. Signals were measured and deconvoluted using a KLAS-100 spectrophotometer (Heinz Walz GmbH) as previously described (Rott et al., 2011).

### Thylakoid isolation

Leaves from 6- to 8-week-old *Oenothera* plants were harvested in the beginning of the light phase and shortly placed in ice water. Ten grams of leave material was mixed with 200 mL isolation medium [50 mM HEPES/KOH (pH 7.6), 330 mM sorbitol, 2 mM EDTA, 1 mM MgCl_2_, 5 mM sodium ascorbate] and blended in a Waring blender (five times 5 s each). Then, the homogenate was filtered through a double layer of mull and Miracloth (Merck), and the filtrate centrifuged at 5,000*g* for 5 min at 4°C. Subsequently, the pellet was resuspended with a brush in 40 mL Wash Medium [50 mM HEPES/KOH (pH 7.6), 5 mM sorbitol)], homogenized in a 30 mL potter homogenizer (PYREX) until the chloroplast pellet was dissolved, and centrifuged at 5,000*g*, 5 min, 4°C. The pellet was then again resuspended, homogenized in a potter homogenizer and centrifuged two times as above. Finally, the pellet was resuspended in 5-mL washing medium, layered on top of 85% Percoll solution [85% PBF-Percoll stock solution contains 3% [w/v] PEG 6000, 1% [w/v] BSA, 1% [w/v] Ficoll 400; 330 mM sorbitol, 50 mM HEPEs/KOH (pH7.6), 2 mM EDTA, 1 mM MgCl_2_] and centrifuged at 10,000*g* for 5 min at 4°C. Thylakoids were washed once with wash medium (5000*g*, 5 min, 4°C) and resuspended in storage buffer [50 mM HEPES/KOH (pH 7.6), 10% [v/v] glycerol, 5 mM MgCl_2_, 100 mM sorbitol], frozen in liquid N_2_, and stored at −80°C until use.

### Protein isolation and sodium dodecyl sulphate-polyacrylamide gel electrophoresis (SDS–PAGE)

Plant total proteins were extracted from 20 mg of frozen ground leaf material homogenized in 100 µL of extraction buffer [25 mM BisTris/HCl (pH 7.0), 20% [v/v] glycerol, 0.25 g·L^−1^ complete protease inhibitor (Roche)]. Membranes were solubilized by addition of 1% [w/v] n-dodecyl-β-d-maltoside (Sigma Aldrich) in the darkness on ice for 5 min. Finally, samples were denatured in sample buffer (62 mM Tris/HCl, pH 6.8, 10% [v/v] glycerol, 2% [v/v] sodium dodecyl sulfate, 0.0025% [w/v] bromphenol blue) for 3 min at 95°C. Equal volumes of crude extracts were loaded on the gel, 20 µL of crude extract corresponds to 100%. SDS–PAGE with 9% [T] separation gels was performed according to (Laemmli, 1970). After electrophoresis, proteins were visualized by Coomassie staining. Separation gels were incubated in staining solution (0.1% [w/v] Coomassie Blue G, 0.1% [w/v] Coomassie Blue R, 45% [v/v] methanol, 9% [v/v] acetic acid) for 10 min under continuous agitation at room temperature. Gels were destained by incubation in 30% [v/v] methanol, 5% [v/v] acetic acid solution.

### Immunoblot analyses

Proteins separated by SDS–PAGE were transferred to polyvinylidene difluoride membranes (0.2 µm, Thermo Scientific) using the standard Towbin buffer (Towbin et al., 1979) and a tank blotting system (Mini Trans-Blot, Bio Rad) according to the supplier recommendation. Primary antibodies produced in rabbit against AtpE (AS10 1586, 1:2,000), PsaD (AS09 461, 1:1,000), PsbD (AS06 146, 1:1,000), and PetA (AS08 306, 1:2,000) were purchased from Agrisera and applied according to the supplier recommendations. Antibodies generated against chloroplast α and β CF1 subunits in spinach (AtpA/B, 1:1,000) were obtained from Richard Berzborn, University of Bochum, Germany. Antiserum 119 against AtpA/AtpB from spinach was generated similarly as described in (Berzborn, 1980; Otto and Berzborn, 1989). As a control a crude extract of knock-out *ΔatpB* mutant (Hager, 2002) was used. No bands were detected here, confirming a specific detection of AtpA/AtpB (Supplemental Figure 1B). As secondary antibody, an anti-rabbit IgG peroxidase conjugate was used (AS09 602, Agrisera, 1:40,000). Immunochemical detection was performed using the ECL system (GE Healthcare) according to the supplier’s recommendation and documented with Syngene G:BOX Chemi XT4 (SynOptics).

### Sample preparation for LC–MS/MS

For in-solution digestion, thylakoids from *Oe*-WT and I-iota (50 µg each) were solubilized in UTU buffer (6 M urea/2 M thiourea, pH 8.0) and subsequently incubated in reduction buffer (1-µg·µL^−1^ DTT in ddH_2_O) for 1 h at 50°C followed by incubation with alkylation buffer (55 mM iodoacetamide in ddH_2_O) in the dark for 30 min at room temperature. The samples were then treated with endoproteinase Lys-C (0.5 µg·µL^−1^ 10 mM Tris/HCl, pH 8.0) for 4 h at room temperature and digested in 0.4 µg·µL^−1^ of trypsin (both purchased from Promega) overnight at room temperature. BSA was used as a control and treated identically. Digestion was stopped by addition of TFA (0.2% [v/v] final concentration). Peptides were purified using Zip tip C18 pipette tips (Millipore) according to the manufacturer’s instructions.

For in-gel digestion, SDS–PAGE-separated and Coomassie-stained protein bands were excised from the gel and destained using a mixture of 40% [v/v] acetonitrile and 60% 50 mM [v/v] NH_4_HCO_3_ at 37°C under continuous agitation. Gel pieces were dried by vacuum centrifugation and incubated with trypsin (modified sequencing grade, Roche) in 50 mM NH_4_HCO_3_ or Glu-C (sequencing grade, Promega) in 50 mM Tris/PO_4_, pH 7.8 for 12–16 h at 37°C. Proteolytic peptides were extracted by repetitive incubation with acetonitrile, 5% [v/v] formic acid, and again acetonitrile. The combined supernatants were dried by vacuum centrifugation, and peptides from were purified using Zip tip C18 pipette tips (Millipore) according to the manufacturer’s instructions.

### LC–MS/MS

Samples generated by in-solution digestion for quantification of AtpA, AtpB, and AtpE in *Oe*-WT and I-iota were subjected LC**–**MS/MS with free labeling approach. The peptides were resuspended in 2% [v/v] acetonitrile, 0.1% [v/v] TFA and injected using nanoflow high-performance liquid chromatography (HPLC, Proxeon Biosystems) into the analytical column (length: 12 cm, diameter: 75 μm) filled with C18 (Reprosil C18; Dr Maisch GmbH). Peptides were eluted from the column in a 90-min linear gradient of 5%–80% [v/v] acetonitrile at a flow rate of 250 µL·min^−1^. Samples were analyzed using the selected reaction monitoring method. Fragmentation and detection were performed with a TSQ Quantum UltraTM Triple Stage Quadrupole Mass Spectrometer (ThermoFisher Scientific). Specific peptide sequences unique for the target proteins (AtpA, AtpB, AtpE) were created using the Pinpoint software (version 1.0, Thermo Scientific) and are listed in Supplemental Table 4. Peptides obtained from MS were analyzed with the Pinpoint software (Thermo Scientific).

Samples generated by in-gel digestion were resuspended in 5% [v/v] acetonitrile and 0.1% [v/v] formic acid. Peptides were separated on a C18 reversed-phase analytical column (Acclaim PepMap100, ThermoFisher Scientific) using an Easy-nLC 1000 liquid chromatography system (ThermoFisher Scientific). Peptides were eluted in a 28-min-long nonlinear 5%–34% [v/v] acetonitrile gradient in 0.1% [v/v] formic acid and 5% [v/v] DMSO at a flow of 300 nL min^−1^. Subsequently, the column was cleaned for 10 min with 85% [v/v] acetonitrile in 0.1% [v/v] formic acid and 5% [v/v] DMSO. Eluted peptides were transferred to an NSI source and sprayed into an Orbitrap Q-Exactive Plus mass spectrometer (ThermoFisher Scientific). The MS was run in positive ion mode. For full MS scans, the following settings were used: resolution: 70,000, AGC target: 3E6, maximum injection time: 100 ms, and scan range: 200 to 2,000 m/z. For dd-MS^2^, the following settings were used: resolution: 1,75,000, AGC target: 1E5, maximum injection time: 50 ms, loop count: 15, isolation window: 4.0 m/z, and NCE: 30. In addition, the following data-dependent settings were used: underfill ratio: 1%, apex trigger: off, charge exclusion: unassigned, 1, 5, 5–8, >8, peptide match: preferred, exclude isotypes: on, and dynamic exclusion: 20.0 s.

The raw files obtained from Xcalibur (Thermo Fisher Scientific) were converted into mgf files using MSConvert from the ProteoWizard portal. The files were analyzed by the Mascot server with an in-house database containing protein sequences from several plant species POTbase MS (Moreno et al., 2018), or a database containing the AtpB sequence and the potential variations in lysine content (summarized in Table 1, for sequences see Supplemental Table 1).

### Dual-luciferase reporter assay

The dual-luciferase reporter plasmid was designed on the basis of the pEK4 vector (Kramer and Farabaugh, 2007). 150 bp from the 5′-region of *atpB* from *N. tabacum* and *O. elata* (GenBank accession numbers Z00044.2 and KT881170.1, respectively) containing the oligoA stretch were cloned into the polylinker of the insertion window of pEK4 using BglII and BamHI restriction sites. Final constructs were transformed into NEBExpress chemically competent *E. coli* cells (C3037I, NEB). Cultures of three individual clones were grown at 37°C in LB medium supplemented with 100 µg mL^−1^ of ampicillin. Upon reaching an OD of 0.4, equal volumes of suspension were collected. Crude extracts were prepared according to (Meydan et al., 2017). The activities of firefly and Renilla luciferases were measured using the dual-luciferase reporter assay system (Promega). Luminescence was followed in 96-well plates in a ClarioStar micro plate reader (BMG Labtech). The translation efficiency was calculated according to (Grentzmann et al., 1998).

### Transmission electron microscopy

*Oenothera* leaf pieces of 2 mm^2^ were fixed with 2.5% [w/v] glutaraldehyde and 2% [w/v] paraformaldehyde in 0.2 M sodium cacodylate buffer (pH 7.4) for at least 4 h at 4°C. Samples were then post-fixed in 1% [w/v] OsO_4_ at 4°C overnight, stained in 2% [w/v] aqueous uranyl acetate for 2 h and dehydrated gradually in 30%, 50%, 70%, 80%, 90%, and 100% [v/v] ethanol followed by washing in 100% [v/v] propylene oxide two times. The samples were then embedded in low-melting Spurr epoxy resin (Agar Scientific), degassed and cured at 65°C for 24 h. Thin sections (60–70 nm) were obtained with a Leica Ultracut UC 6 (Leica Microsystems), mounted on 150-mesh nickel grids, counterstained with uranyl acetate followed by lead citrate, and examined with a transmission electron microscope at 120 kV (EFTEM, Zeiss).

### Statistical analysis

Statistical analysis (*t*-test and one-way analysis of variance (ANOVA) with Holm–Sidak post-hoc testing) were performed with SigmaPlot14. Type of statistical tests used and replicate numbers are as indicated in the figure legends.

### Accession numbers

Chloroplast genome sequence data from this article can be found in the GenBank data libraries under accession numbers EU262891.2 (*Oenothera parviflora* strain atrovirens Standard), KT881170.1 (*Oenothera elata* subsp. *hookeri* strain de Vries), Z00044.2 (*N. tabacum*), NC_000932.1 (*Arabidopsis thaliana*), NC_031333.1 (*Oryza sativa* cultivar TN1), NC_008096.2 (*Solanum tuberosum*), NC_001666.2 (*Zea mays*). The proteomic datasets have been deposited in the ProteomeXchange Consortium (http://www.proteomexchange.org) via the PRIDE partner repository with the dataset identifier PXD020246.

## Supplemental data

The following materials are available in the online version of this article.

**Supplemental Figure 1**. Original blots and PCR confirming homoplasmy.

**Supplemental Figure 2**. Graphical output of predicted RNA secondary structure in a set of *atpB* aligned sequences.

**Supplemental Figure 3**. LC–MS/MS fragmentation spectrum of the targeted AtpB peptide in *Nt*-WT.

**Supplemental Figure 4**. cDNA analysis of *Oenothera* wild-type (*Oe*-WT) and I-iota (+1A) tissue from different developmental stages.

**Supplemental Figure 5**. cDNA analysis to test for RNA polymerase stuttering in *atpB*.

**Supplemental Table 1**. Alternative amino acid sequences of AtpB in *Oenothera* and tobacco as caused by genetic mutations or reading frame shifts at the slip site used for the database generation.

**Supplemental Table 2**. Dual-luciferase constructs generated for analysis of frameshift efficiency in *Oenothera* and tobacco *atpB* sequences tested in *E. coli*.

**Supplemental Table 3**. Primers used in this study.

**Supplemental Table 4**. Peptides from ATP synthase subunits of Oenothera and bovine serum albumin (BSA) detected by selected reaction monitoring (SRM).

**Supplemental Data Set 1**. *t*-test and one-way ANOVA reports generated by SigmaPlot14 software.

## Supplementary Material

koab050_Supplementary_Data

## Notes

I.M., A.Z., A.M. performed protein analysis (immunoblot and samples preparation for MS). E.H.M. and W.S. performed MS analysis. I.M., M.R, L.Y.R., and S.R. generated and identified tobacco transplastomic lines. M.A.S. performed photosynthesis analysis and measured the activity of the ATP synthase. All authors analyzed and discussed the data. I.M. and S.G. designed the study. I.M. wrote the manuscript with significant contribution from S.G., M.A.S., and R.B. All authors approved the final version.

**Author responsibility**

The author responsible for distribution of materials integral to the findings presented in this article in accordance with the policy described in the Instructions for Authors (https://academic.oup.com/plcell) is: Stephan Greiner (greiner@mpimp-golm.mpg.de).
