# Supplementary material for: Correction of frameshift mutations in the atpB gene by translational recoding in chloroplasts of Oenothera and tobacco
Source: Plant Cell. 2021 Feb 9;33(5):1682–705. doi: 10.1093/plcell/koab050 (PMC8254509; doi:10.1093/plcell/koab050)
Supplement: koab050_Supplementary_Data [file koab050_supplementary_data.zip › tpc.00686.2020-s01.pdf]

Supplemental Data. Malinova et al. (2021). Plant Cell.

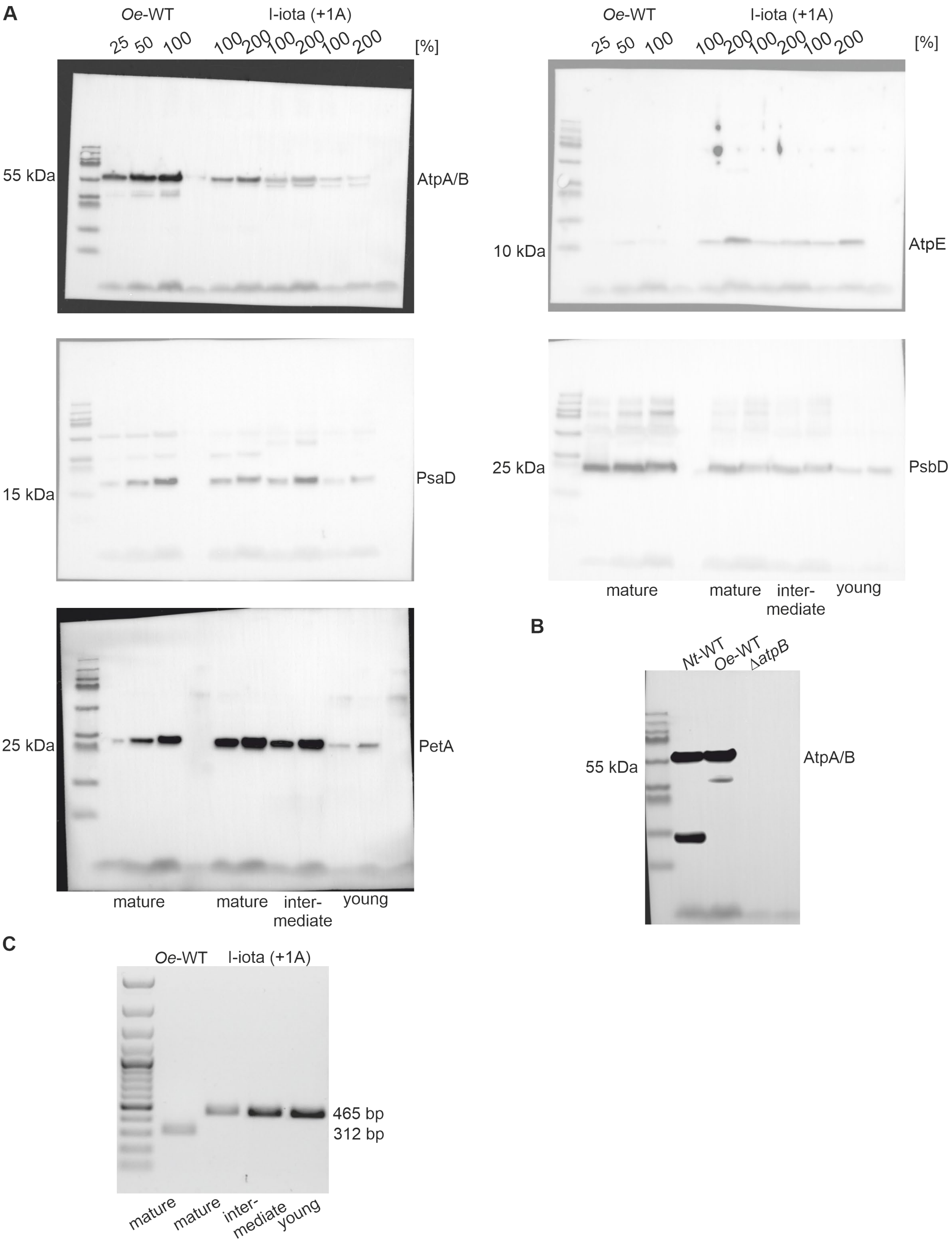

Supplemental Figure 1. Original blots and PCR confirming homoplasmy. (Supports Figure 2).

A. Original immunoblot membranes are shown. Leaves were harvested from 6-8-week-old plants. For the I-iota mutant, leaf tissues of different ages were analyzed (young: top leaves of the rosette, intermediate: mottled leaves from the middle of the rosette, mature: greenish part of leaves from the base of the rosette; see Figure 1A). Equal fresh weight (20 mg, for details see Material and Methods) were used for crude extract preparation. Samples were loaded based on equal sample volume (20 µl=100 %) **B.** Validation of the AtpA/B antibody. The tobacco □*atpB* knock-out mutant (Hager, 2002) was used as a control. **C.** PCR confirming homoplasmy of homogenized leaf material used for the SDS PAGE and immunoblot in **A** (*clpP* marker [details in Figure 1B]).

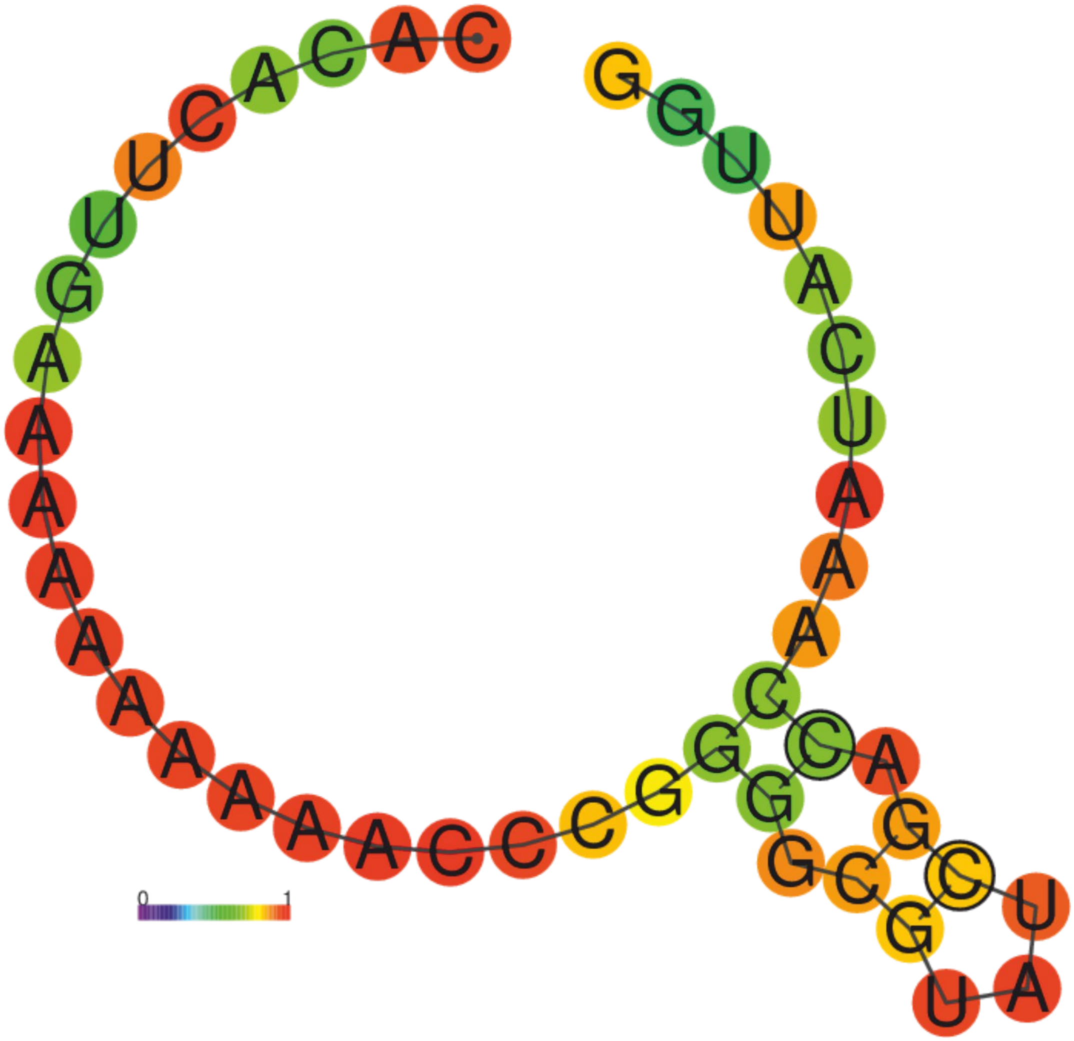

Supplemental Figure 2. Graphical output of predicted RNA secondary structure in a set of aligned sequences. (Supports Figure 4).
5’ parts of the *atpB* coding region of *Arabidopsis thaliana*, *Oryza sativa*, *Solanum tuberosum*, *Zea mays*, *Oenothera elata*, and *Nicotiana tabacum* were analyzed by the RNAalifold web server. Color coding reflects the base-pair probability.

Nt-WT

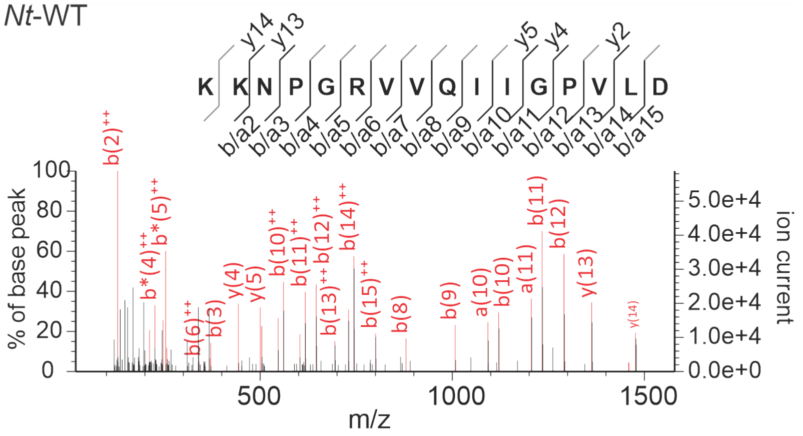

Supplemental Figure 3. LC-MS/MS fragmentation spectrum of the targeted AtpB peptide in *Nt*-WT. (Supports Figure 5).

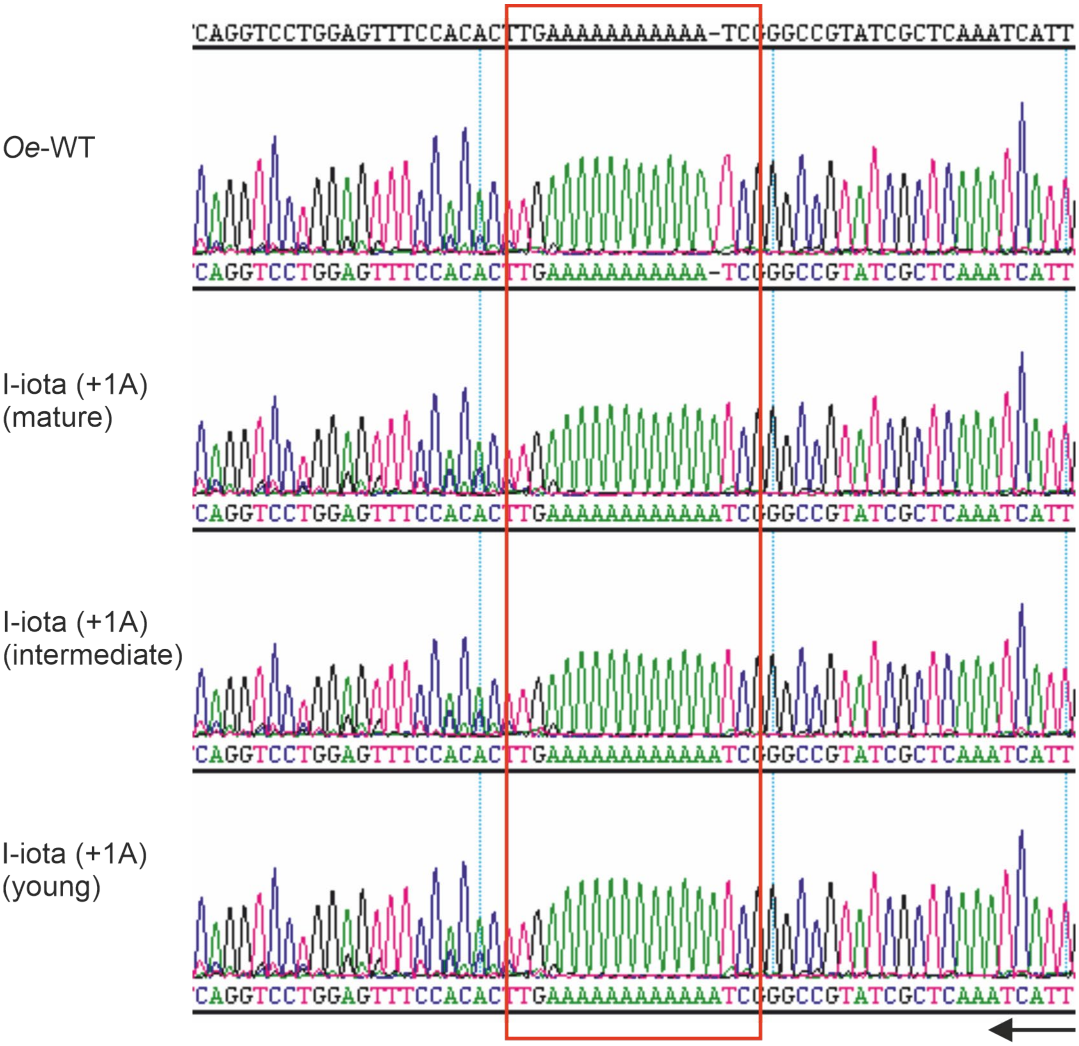

Supplemental Figure 4. cDNA analysis of *Oenothera* wild type [*Oe*-WT) and I-iota (+1A) tissue from different developmental stages. (Supports Figure 10).
The amplified cDNA population was sequenced, and typical Sanger sequencing chromatograms are presented. The sequence containing the oligoA stretch is boxed in red. Black arrows indicate the direction of the sequencing reaction. Typical chromatograms are shown. cDNA analyses were repeated at least once with separate batches of plants. For definition of the developmental stage see Supplemental Figure 1.

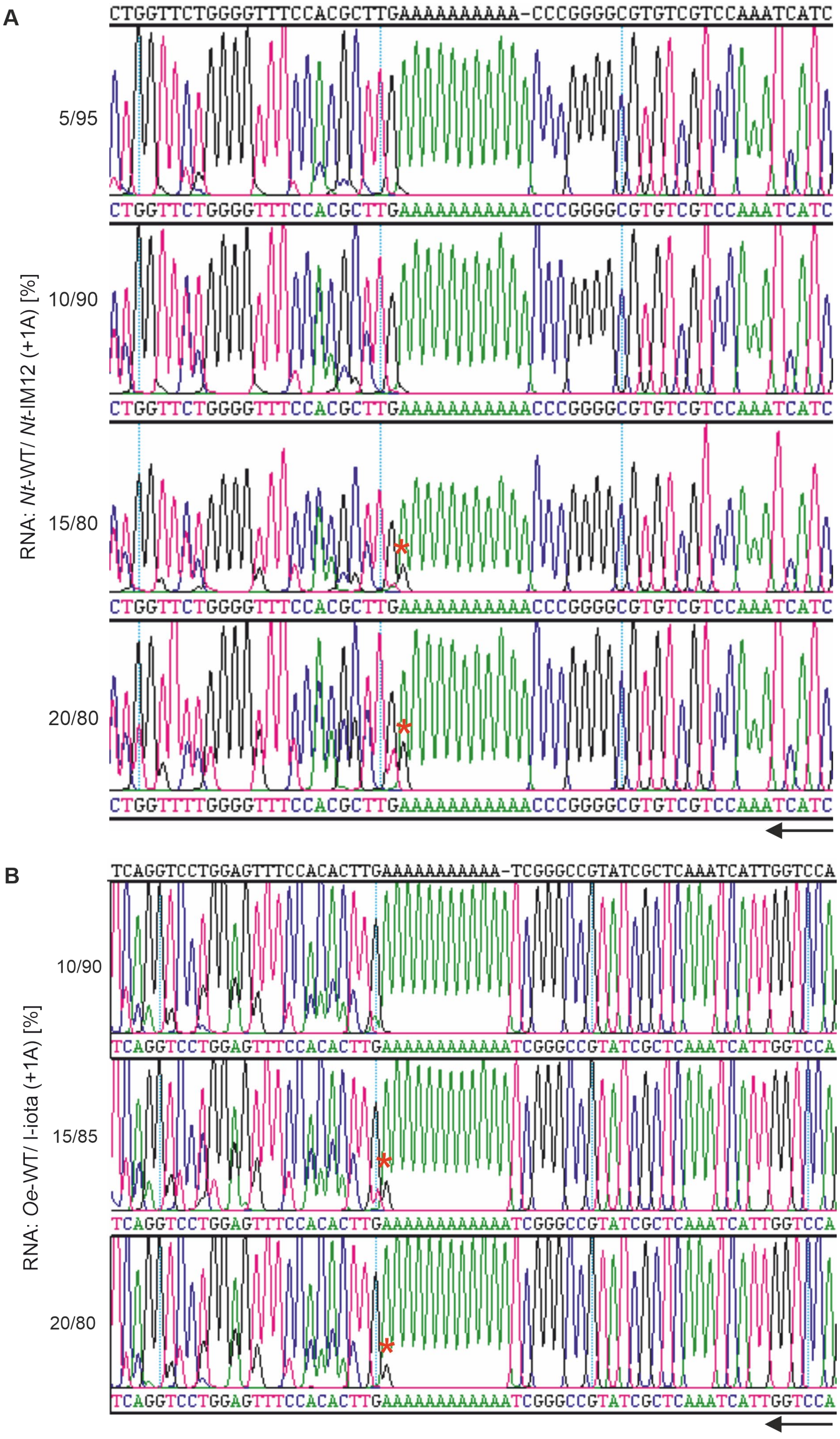

Supplemental Figure 5. cDNA analysis to test for RNA polymerase stuttering in *atpB*. (Supports Figure 10).

Sensitivity test for the detection of RNA polymerase stuttering. RNA was extracted from the tobacco wild type (*Nt*-WT) and the *Nt*-IM12 (+1A) transplastomic line **(A)**, *Oenothera* wild type and I-iota (+1A) mutant **(B)**, and the RNA samples were mixed in different ratios

(indicated in %). cDNA was the synthesized, amplified and sequenced. Red asterisks indicate the additional peak, corresponding to the spiked-in wild-type RNA. Black arrows indicate the direction of the sequencing reaction. **A-B**. Typical chromatograms are shown. cDNA analyses were repeated at least once with separate batches of plants.

Supplemental Table 1. **Alternative amino acid sequences of AtpB in *Oenothera* and tobacco as caused by genetic mutations or reading frame shifts at the slip site used for the database generation.** The lysine (K) stretch affected by the mutation is highlighted in grey.

Species	Protein variant	Amino acid sequence
Oenothera	AtpB_*Oe*_WT	MRINPTTSGPGVSTLEKKKSGRIAQIIGPVLDVTFPPGKMPNIYNALVVKGRDTGGQEINVTCEVQQLLGNNRVRAVAMSATD<br>GLTRGMEVIDTGAPLSVPVGGATLGRIFNVLGEPVDELGPVDTRTTSPIHRSAPAFIQLDTKLSIFETGIKVVDLLAPYRRGGKIG<br>LFGGAGVGKTVLIMELINNIAKAHGGVSVFGGVGERTREGNDLYMEMKESGVINEQNIAESKVALVYGQMNEPPGARMRVGL<br>TALTMAEYFRDVNKQNVLLFIDNIFRFVQAGSEVSALLGRMPSAVGYQPTLSTEMGSLQERITSTKAGSITSIQAVYVPADDLTD<br>PAPATTFAHLDATTVLSRGLAAKGIYPAVDPLDSTSTMLQPRIVGDEHYETAQRVKETLQRYKELQDIISILGLDELSEEDRLTVA<br>RARKIERFLSQPFFVAEVFTGSPGKYVGLAETIRGFKLILSGELDGLPEQAFYLVGTIDEATAKAANLEMESDLKK
	AtpB_*Oe*_I-iota	MRINPTTSGPGVSTLEKKKIGPYRSNHWSSTGCNLSPGEDA
	AtpB_*Oe*_2K	MRINPTTSGPGVSTLEKKSGRIAQIIGPVLDVTFPPGKMPNIYNALVVKGRDTGGQEINVTCEVQQLLGNNRVRAVAMSATDGLTR<br>GMEVIDTGAPLSVPVGGATLGRIFNVLGEPVDELGPVDTRTTSPIHRSAPAFIQLDTKLSIFETGIKVVDLLAPYRRGGKIGLFGGAG<br>VGKTVLIMELINNIAKAHGGVSVFGGVGERTREGNDLYMEMKESGVINEQNIAESKVALVYGQMNEPPGARMRVGLTALTMAEY<br>FRDVNKQNVLLFIDNIFRFVQAGSEVSALLGRMPSAVGYQPTLSTEMGSLQERITSTKAGSITSIQAVYVPADDLTDPAPATTFAHLD<br>ATTVLSRGLAAKGIYPAVDPLDSTSTMLQPRIVGDEHYETAQRVKETLQRYKELQDIISILGLDELSEEDRLTVARARKIERFLSQPFF<br>VAEVFTGSPGKYVGLAETIRGFKLILSGELDGLPEQAFYLVGTIDEATAKAANLEMESDLKK
	AtpB_*Oe*_4K	MRINPTTSGPGVSTLEKKKKSGRIAQIIGPVLDVTFPPGKMPNIYNALVVKGRDTGGQEINVTCEVQQLLGNNRVRAVAMSATDGL<br>TRGMEVIDTGAPLSVPVGGATLGRIFNVLGEPVDELGPVDTRTTSPIHRSAPAFIQLDTKLSIFETGIKVVDLLAPYRRGGKIGLFGGA<br>GVGKTVLIMELINNIAKAHGGVSVFGGVGERTREGNDLYMEMKESGVINEQNIAESKVALVYGQMNEPPGARMRVGLTALTMAE<br>YFRDVNKQNVLLFIDNIFRFVQAGSEVSALLGRMPSAVGYQPTLSTEMGSLQERITSTKAGSITSIQAVYVPADDLTDPAPATTFAHL<br>DATTVLSRGLAAKGIYPAVDPLDSTSTMLQPRIVGDEHYETAQRVKETLQRYKELQDIISILGLDELSEEDRLTVARARKIERFLSQPF<br>FVAEVFTGSPGKYVGLAETIRGFKLILSGELDGLPEQAFYLVGTIDEATAKAANLEMESDLKK
tobacco	AtpB_*Nt*_wt	MRINPTTSGSGVSTLEKKNPGRVVQIIGPVLDVAFPPGKMPNIYNALVVQGRDSVGQPINVACEVQQLLGNNRVRAVAMSATEGLT<br>RGMEVIDTGAPISVPVGGATLGRIFNVLGEPVDNLGPVDTSTTSPIHRSAPAFIQLDTKLSIFETGIKVVDLLAPYRRGGKIGLFGGAGV<br>GKTVLIMELINNIAKAHGGVSVFGGVGERTREGNDLYMEMKESGVINEENIAESKVALVYGQMNEPPGARMRVGLTALTMAEYFRD<br>VNEQDVLLFIDNIFRFVQAGSEVSALLGRMPSAVGYQPTLSTEMGSLQERITSTKEGSITSIQAVYVPADDLTDPAPATTFAHLDATTVL<br>SRGLAAKGIYPAVDPLDSTSTMLQPRIVGEEHYETAQRVKQTLQRYKELQDIIAILGLDELSEEDRLLVARARKIERFLSQPFFVAEVFT<br>GSPGKYVGLAETIRGFQLILSGELDGLPEQAFYLVGNIDEATAKAMNLEMESNLKK
	AtpB_*Nt*_IM12 truncated	MRINPTTSGSGVSTLEKKKPGACRPNHRSGTRCSLSPGQDAEYL

Supplemental Table 1. (continued)

Species	Protein variant	Amino acid sequence
tobacco	AtpB_*Nt*_IM14 truncated	MRINPTTSGSGVSTLEKKKTRGVSSKSSVRY
	AtpB_*Nt*_IM16 truncated	MRINPTTSGSGVSTLEKKTRGVSSKSSVRY
	AtpB_*Nt*_1K	MRINPTTSGSGVSTLEKNPGRVVQIIGPVLDVAFPPGKMPNIYNALVVQGRDSVGQPINVACEVQQLLGNNRVRAVAMSATEGLTR<br>GMEVIDTGAPISVPVGGATLGRIFNVLGEPVDNLGPVDTSTTSPIHRSAPAFIQLDTKLSIFETGIKVVDLLAPYRRGGKIGLFGGAGV<br>GKTVLIMELINNIAKAHGGVSVFGGVGERTREGNDLYMEMKESGVINEENIAESKVALVYGQMNEPPGARMRVGLTALTMAEYFR<br>DVNEQDVLLFIDNIFRFVQAGSEVSALLGRMPSAVGYQPTLSTEMGSLQERITSTKEGSITSIQAVYVPADDLTDPAPATTFAHLDAT<br>TVLSRGLAAKGIYPAVDPLDSTSTMLQPRIVGEEHYETAQRVKQTLQRYKELQDIIAILGLDELSEEDRLLVARARKIERFLSQPFFVA<br>EVFTGSPGKYVGLAETIRGFQLILSGELDGLPEQAFYLVGNIDEATAKAMNLEMESNLKK
	AtpB_*Nt*_3K	MRINPTTSGSGVSTLEKKKNPGRVVQIIGPVLDVAFPPGKMPNIYNALVVQGRDSVGQPINVACEVQQLLGNNRVRAVAMSATEGL<br>TRGMEVIDTGAPISVPVGGATLGRIFNVLGEPVDNLGPVDTSTTSPIHRSAPAFIQLDTKLSIFETGIKVVDLLAPYRRGGKIGLFGGAG<br>VGKTVLIMELINNIAKAHGGVSVFGGVGERTREGNDLYMEMKESGVINEENIAESKVALVYGQMNEPPGARMRVGLTALTMAEYF<br>RDVNEQDVLLFIDNIFRFVQAGSEVSALLGRMPSAVGYQPTLSTEMGSLQERITSTKEGSITSIQAVYVPADDLTDPAPATTFAHLDAT<br>TVLSRGLAAKGIYPAVDPLDSTSTMLQPRIVGEEHYETAQRVKQTLQRYKELQDIIAILGLDELSEEDRLLVARARKIERFLSQPFFVAE<br>VFTGSPGKYVGLAETIRGFQLILSGELDGLPEQAFYLVGNIDEATAKAMNLEMESNLKK

Supplemental Table 2. Dual-luciferase constructs generated for analysis of frameshift efficiency in *Oenothera* and tobacco *atpB* sequences tested in *E. coli*. Insertions and deletions are denoted in red and blue, respectively. Adenines replaced by guanines are highlighted in yellow.

Construct	Oligo(A) stretch	Test sequence length [bp]	Species
**pEK4-*Oe*-WT**	AAAAAAAAAAA	150	*Oenothera*
**pEK4-I-iota (+1A)**	AAAAAAAAAAAA	150**+1**	
**pEK4-*Nt*-WT**	AAAAAAAAAA	150	tobacco
**pEK4-*Nt*-IM11 (AAG)**	AGAAGAAGAA	150	
**pEK4-*Nt*-IM12 (+1A)**	AAAAAAAAAAA	150**+1**	
**pEK4-*Nt*-IM13 (AAG+1)**	AGAAGAAGAAA	150**+1**	
**pEK4-*Nt*-IM14 (+2A)**	AAAAAAAAAAAA	150**+2**	
**pEK4-*Nt*-IM15 (AAG+2)**	AGAAGAAGAAAA	150**+2**	
**pEK4-*Nt*-IM16 (-1A)**	AAAAAAAAA-	150**-1**	
**pEK4-*Nt*-IM17 (AAG-1A)**	AGAAGAAGA-	150**-1**	
**pEK4-*Nt*-IM18 (-2A)**	AAAAAAAA--	150**-2**	
**pEK4-*Nt*-IM19 (AAG-2A)**	AGAAGAAG--	150**-2**	

Supplemental Table 3. Primers used in this study. All primer sequences are given in the 5’ to 3’ direction. Restriction sites are underlined.

Name	Primer sequence	Species	Comments
clpP_IvsIV_for	ATACACTACAATAAAGAAGGAGCAAGTG	*Oenothera*	Identification of *O. parviflora* (plastome
clpP_IvsIV_rev	ATCAGCCCGAGTCCGATTATACTC	*Oenothera*	IV) and *O. elata* (plastome I) chloroplasts
rbcLOPI-2	AGAGTTCAGGTTCGAATTCC	*Oenothera*	Amplification of 5’ part of *atpB*
HatpBrev2	AACACTTAGAGGAGCTCCCG	*Oenothera*	
polyA10_3GAA_for	GGTTTCCACGCTTGAGAAGAAGAACCCGGGG	*Nicotiana tabacum*	Site-directed mutagenesis
polyA10_3GAA_rev	CCCCGGGTTCTTCTTCTCAAGCGTGGAAACC	*Nicotiana tabacum*	(pIM11)
polyA_11thA_for	GCTTGAAAAAAAAAAACCCGGGGCGTGTCGT	*Nicotiana tabacum*	Site-directed mutagenesis
polyA_11thA_rev	ACGACACGCCCCGGGTTTTTTTTTTTCAAGC	*Nicotiana tabacum*	(pIM12)
polyA11_3GAA_for	GGTTTCCACGCTTGAGAAGAAGAAACCCGGG	*Nicotiana tabacum*	Site-directed mutagenesis
polyA11_3GAA_rev	CCCGGGTTTCTTCTTCTCAAGCGTGGAAACC	*Nicotiana tabacum*	(pIM13)
atpB(12A)+2_for	TTTCCACGCTTGAAAAAAAAAAAACCCGGGGCGTGT	*Nicotiana tabacum*	Site-directed mutagenesis
atpB(12A)+2_rev	ACACGCCCCGGGTTTTTTTTTTTTCAAGCGTGGAAA	*Nicotiana tabacum*	(pIM14)
atpB(GAA12)_for	GGGGTTTCCACGCTTGAGAAGAAGAAAACCCGGGGCGTGTC	*Nicotiana tabacum*	Site-directed mutagenesis
atpB(GAA12)_rev	GACACGCCCCGGGTTTTCTTCTTCTCAAGCGTGGAAACCCC	*Nicotiana tabacum*	(pIM15)
atpB(9A)-1(-4)_for	GGTTTCCACGCTTGAAAAAAAAACCCGGGGCGTGT	*Nicotiana tabacum*	Site-directed mutagenesis
atpB(9A)-1(-4)_rev	ACACGCCCCGGGTTTTTTTTTCAAGCGTGGAAACC	*Nicotiana tabacum*	(pIM16)
atpB(GAA9)_for	GGTTCTGGGGTTTCCACGCTTGAGAAGAAGACCCGGGGCGTG	*Nicotiana tabacum*	Site-directed mutagenesis
atpB(GAA9)_rev	CACGCCCCGGGTCTTCTTCTCAAGCGTGGAAACCCCAGAACC	*Nicotiana tabacum*	(pIM17)
atpB(8A)_for	GTTTCCACGCTTGAAAAAAAACCCGGGGCGTG	*Nicotiana tabacum*	Site-directed mutagenesis
atpB(8A)_rev	CACGCCCCGGGTTTTTTTTCAAGCGTGGAAAC	*Nicotiana tabacum*	(pIM18)
atpB(GAA8)_for	TTCTGGGGTTTCCACGCTTGAGAAGAAGCCCGGGGCGTG	*Nicotiana tabacum*	Site-directed mutagenesis
atpB(GAA8)_rev	CACGCCCCGGGCTTCTTCTCAAGCGTGGAAACCCCAGAA	*Nicotiana tabacum*	(pIM18)
atpB_BglII_f	CGTATAAGATCTATGAGAATCAATCCTACTAC	*Nicotiana / Oenothera*	Cloning of 5’ 150 bp of *atpB* into pEK4
atpB_BamHI_r	CACTTCGGATCCTTTAACGACCAGAGCGTTATA	*Oenothera*	Cloning of 5’ 150 bp of *atpB* into pEK4
Nt_atpB_BamHI	CACTTCGGATCCTTGAACTACCAGAGCGTTAT	*Nicotiana tabacum*	Cloning of 5’ 150 bp of *atpB* into pEK4

Supplemental Table 3. (continued)

Name	Primer sequence	Species	Comments
atpB_start	ATGAGAATCAATCCTACTACTT	*Nicotiana / Oenothera*	PCR amplification
atpB_Oe_RT_cDNA	TCTTCAAATCACTCTCCATTTCTAAGTTCG	*Oenothera*	Gene-specific primer for cDNA synthesis
atpB_Nt_RT_cDNA_2	CCATCTAATTCTCCGGAAAGGATCAATTG	*Nicotiana tabacum*	Gene-specific primer for cDNA synthesis
HatpBrev	CTTGCTCATTAACATCTCG	*Nicotiana tabacum*	PCR amplification
aad2_pMJR10_f	CTCGCCGCGTTGTTTCATCAAGCC	*Nicotiana tabacum*	PCR test for identification of
'-35atpE	CTTGTAAAGAACCCATTTCGG	*Nicotiana tabacum*	transplastomic lines
patpB_pMJR10_f	CTCAAGATTCTGATTCATCCACTTG	*Nicotiana tabacum*	Sequencing of transplastomic tobacco lines
atpB.P1	CAAACCAACTCTCATACGAGC	*Nicotiana tabacum*	Probe amplification for RFLP

atpB1_pMJR10_f	GTGCTACAGAGGGTCTAACGAG	*Nicotiana tabacum*

Supplemental Table 4. Peptides from ATP synthase subunits of *Oenothera* and bovine serum albumin (BSA) detected by selected reaction monitoring (SRM).

Protein	Peptide	Mass/ charge ratio (m/z)
**AtpA**	ASSVAQVVNALQER	736.397
	EAYPGDVFYLHSR	777.373
	ELIIGDR	408.235
	FLDELR	396.716
	IAQIPVSEAYLGR	708.896
	IFTEEAQALLK	631.853
	IVNTGTVLQVGDGIAR	806.954
	KPQFEEIISSTK	703.88
	LIESPAPGIISR	626.867
	NVGVVLMGDGLLIQEGSSVK	1008.046
	QMSLLLR	430.755
**AtpB**	AANLEMESDLK	610.795
	AHGGVSVFGGVGER	664.839
	AVAMSATDGLTR	596.803
	ESGVINEQNIAESK	759.376
	FVQAGSEVSALLGR	717.391
	GIYPAVDPLDSTSTMLQPR	1031.02
	GMEVIDTGAPLSVPVGGATLGR	1049.054
	IAQIIGPVLDVTFPPGK	883.017
	IFNVLGEPVDELGPVDTR	985.515
	INPTTSGPGVSTLEK	750.899
	LSIFETGIK	504.292
	MPNIYNALVVK	631.352
	VALVYGQMNEPPGAR	801.409
	VGLTALTMAEYFR	736.384
	VVDLLAPYR	523.306
**AtpE**	GSDIDPQEAQETLGLAEANFR	1131.038
	IGNNEITILVNDAEK	821.936
	IVWDSEVK	488.261
	QNGQWLTMALMGGFAR	890.935
	QTIEANLALR	564.822
	TLNLCVLTPNR	622.345
	TLNLC[Carboxyamidomethyl]VLTPNR	650.856
	VEAINVIS	422.742
**BSA**	DDSPDLPK	443.711
	DLGEEHFK	487.733
	HLVDEPQNLIK	653.362
	LGEYGFQNALIVR	740.401
	LVNELTEFAK	582.319
	LVTDLTK	395.239
	QTALVELLK	507.813
	SEIAHR	356.69
	TPVSEK	330.682
	VPQVSTPTLVEVSR	756.425
	YLYEIAR	464.25
