# Supplementary material for: Correction of frameshift mutations in the atpB gene by translational recoding in chloroplasts of Oenothera and tobacco
Source: Plant Cell. 2021 Feb 9;33(5):1682–705. doi: 10.1093/plcell/koab050 (PMC8254509; doi:10.1093/plcell/koab050)
Supplement: koab050_Supplementary_Data [file koab050_supplementary_data.zip › tpc.00686.2020-s04.pdf]

Correction of frameshift mutations in the *atpB* gene by translational recoding in chloroplasts of *Oenothera* and tobacco

Irina Malinova, Arkadiusz Zupok, Amid Massouh, Mark Aurel Schöttler, Etienne H. Meyer, Liliya Yaneva-Roder, Witold Szymanski, Margit Rößner, Stephanie Ruf, Ralph Bock, Stephan Greiner

Corresponding author: Irina Malinova malinova@mpimp-golm.mpg.de

Review timeline:

**TPC2020-RA-00686**	Submission received:	Sept. 11, 2020
	1st Decision:	Oct. 28, 2020 *Accept with minor revisions*
**TPC2020-RA-00686R1**	1st Revision received:	Dec. 10, 2020
	2nd Decision:	Dec. 31, 2020 *acceptance pending, sent to science editor*
	Final acceptance:	Feb. 2, 2021

REPORT: (The report shows the major requests for revision and author responses. Minor comments for revision and miscellaneous correspondence are not included. The original format may not be reflected in this compilation, but the reviewer comments and author responses are not edited, except to correct minor typographical or spelling errors that could be a source of ambiguity.)

TPC2020-RA-00686 1st Editorial decision – *Accept with minor revisions* Oct. 28, 2020

On the basis of the advice received, the board of reviewing editors would like to accept your manuscript for publication in The Plant Cell. This acceptance is contingent on revision based on the comments of our reviewers. In particular, please consider the following:

The reviewers found this to be an interesting and important contribution to the literature. They have made a number of comments for revision, but most should be easy to address. Please address all comments to the best of your ability as you see fit to help clarify and strengthen the presentation of results and conclusions.

-- Reviewer comments:

[Reviewer comments shown below with author responses.]

TPC2020-RA-00686R1 1st Revision received Dec. 10, 2020

Reviewer comments and **author responses**:

Reviewer #1 (Comments for the Author):

In their MS, Malinova et al. describe the analysis of an A-insertional mutation in the plastidial atpB gene encoding an essential component of the ATP synthase enzyme. Mutant seedlings show a variegate/ mottled leaf phenotype and altered plastid biogenesis. Using biochemical and genetic analyses the authors linked the insertional mutation to a translational recoding in the chloroplasts of Oenothera and tobacco plants expressing various atpB variants in their plastids. Accordingly, although such mutations are expected to result with truncated nonfunctional gene products, a full-length AtpB protein is apparent in the both plant's plastids. This research provides with important insights into gene expression and translational activities in land plant organelles. Overall, I find the MS of a high quality. It is well written, clear and concise. The authors have produced a comprehensive dataset and a detailed description of the mechanistic properties of ribosomal frameshifting / recoding in Oenothera "in vivo", as well as by "in vitro" approach in tobacco, using transplastomics plants. The demonstration of this phenomenon in both plant species suggest that these observations are generally applied to angiosperms. The figures and tables are also generally of high quality. This reviewer thinks that the plant community would strongly benefits from this research study when the MS and its

data therein go public. I have some, mostly minor, comments/suggestions to the authors that if followed could potentially improve the reading of this insightful manuscript.

Specific comments to the authors
1. abstract section and following parts of the MS: "single adenine insertion in an oligoA stretch of atpB". I find the term "oligoA" quite confusing in the context of an Adenosine insertion. I would suggest instead using a "long Adenosine-stretch [or poly(A)] present in the coding region".

> **Response: We have changed “single adenine insertion in an oligoA stretch of atpB” for “a single adenine insertion in an oligoA stretch [11A] of *atpB* coding region” (new lines 33 and 34).**

2. Results p.6 : "tests for homoplasmy in green and mottled tissue". Some statistics should be provided here.

> **Response: We do not think that statistic is appropriated here. Linkage of the I-iota mutation of the mottled phenotype is absolute, i.e. *p* = 1. All variegated I-iota samples were tested for homoplasmy before further analysis. We have stated this in the figure legends and, when applicable, and in the main text (new lines 176 and 177).**

3. Results p. 7 : Although Sears and Herrmann (1985) suggested an unexpected high molecular band, and the fact that early reports indicate that in angiosperms chloroplasts the start codon of atpE overlaps with the stop codon of atpB suggesting that atpE may be translationally coupled to atpB (Zurawski et al 1982; Gatenby et al 1989), in reality and in further support of the authors data, ribosome footprint profiling show evidence that atpE translation seems independent of atpB translation and that atpE translation is under the control of its own cis-acting elements in the atpB coding region (Zoschke et al., 2013; Zoschke and Barkan 2015).

> **Response: We agree with the reviewer’s comment. We have modified the text and added the references (new lines 183-184 and 188-190).**

4. Figure 1 - in panel 'b' remove the sizes from the blot and indicate the band sizes by arrows and weiwghts outsode the gel. Panel c - the letters are too small and hard to read

> **Response: We have modified the Figure 1 accordingly.**

5. Results p. 7, p. 8 and Fig 2b and Fig s1: “...suggesting that functional AtpB accumulates over time” AND ...” Taken together, these data strongly suggest partial compensation”. It is quite obvious that the authors show that the phenotypes they see are indeed related to a translational recoding (also supported by the transplastomic data at least). But, regarding the blots – it remains possible that the data may relates to cross reactivity between AtpA and AtpB subunits or maybe AtpB of the mitochondrial isoform?! The authors state in the ‘materials and methods” section that they use polyclonal antibodies for AtpB and AtpE (Agrisera). I saw that the agrisrea anti-AtpB abs cross react with both mito’s and clps (AS05 085-10). Not sure about the crossreactivity of AtpE which shows multiple bands in Fig S1. The authors need to address these in more detail.
Another possibility to be considered is the use of native gels and/or in-gel activity assays to see the status of holo-ATP-synthase enzyme in their plant lines.

> **Response: To clarify this point we used an AtpA/B antibody from the R. Berzborn collection. The collection is a legacy of the lab of Professor Herrmann and contains many references sera from the pioneering times photosynthesis research. This present antibody was generated against purified spinach ATP synthase subunits and no bands were detected in a $\Delta atpB$ knock-out mutant (Supplemental Figure 1B). We also performed SDS PAGE followed by Western Blot using plant crude extracts and probed the membrane with AtpE antibodies from Agrisera. As it shown in Figure 2B and Supplemental Figure 1A no high molecular bands were detected.**

6. ATP synthase enzyme activity: p.8 - "...a residual ATP synthase activity of 29% compared to the wild type...". ~30% is not residual activity. It looks very substantial in light of the expected reduction in protein level. How is this correlated with a major reduction in linear electron transport (fig 3b).

> **Response: The ATP synthase activity was measured as decay kinetic of the proton motive force across the thylakoid membrane during a short interval of darkness, so it is determined per thylakoid membrane. On the other hand, linear electron transport was assessed per leaf area. Chlorophyll content per leaf area decreased to 25 % of the wild-type chlorophyll content, and very likely, thylakoid content behaved in a very similar way. Therefore,**

assuming a reduction in thylakoids per leaf area to 25% of the wild-type level, and a residual ATP synthase activity per thylakoid of 30%, the total ATP synthase activity per area will be less than 10 %. This is well in line with a major reduction in linear electron transport. We have included additional explanation in the main text (new lines 216-226).

7. Figure 2 - panel 'b': the data in this panel should be reorganized and additional blots with other plastidial subunits of (e.g. AtpA and also various other proteins of different complexes) should be applied.

Response: As it was mentioned above, we performed SDS PAGE followed by western blot using leaf crude extracts. We probed the membranes with antibodies against photosynthetic subunits of PSI (PsaD), PSII (PsbD) and cyt-bf (Figure 2B) (new lines 204-208).

8. I wander if Figure 2a, 2b should be included within figure 1 while 2d-f as a separate figure (figure 2) or separate these data into 3 different figures.

Response: We think that our type of figures composition is in a line with the main text. We think that splitting the figure 2 in two and generation of an additional figure will be a bit excessive since we already have 10 figures in the main text.

9. results p. 9 and Figure 3c and 4 - "revealed that the oligoA stretch near the 5' end of the coding region is conserved among higher plants"... the predicted 2nd structure of atpB region is quite speculative with a relatively short stem and a low delta-G. Yet it might be supported by showing that this structure is found in many other different plants as well, as partly shown in fig 4. One way to test this in more detail is the use of RNAalifold webserver (http://rna.tbi.univie.ac.at/cgi-bin/RNAWebSuite/RNAalifold.cgi) that predicts a consensus secondary structure of a set of aligned sequences... (as numerous plastomes are available, the atpB locus of many different monocot and dicot species and maybe to terrestrial plants in general can be assayed to further support this?! Also, I think its not necessary to show the structure also in figure 3.
It would be also interesting to test in silico the effects of A insertions or deletions on the consensus structure.

Response: We thank the reviewer for this valuable comment. We have generated the predicted RNA secondary structure for the sequence alignment (Figure 4A and Supplemental Figure 2), which confirms that the translational recoding could be a mechanism operating in various higher plant (new lines 272-276). We tested also different types of mutations (insertions and deletions). However, this analysis was not conclusive in that A insertions or deletion had no effect on the loop structure. Since this is unsurprising we have not included it into the manuscript.

10. P. 9 results – last paragraph. Correct (Figure 3C, 4B,) to Figures 3C and 4B.

Response: We have changed "(Figure 3C, 4B,)" to (Figure 4, Supplemental Figure 2)" (new lines 275-276).

11. p. 13 remove space after figure 9c ((Figure 8C and 9C_).

Response: We have removed the space after 9C (new line 380).

12. P.10 -check the sense of "Next, we analyzed photosynthesis.." . they may consider 'photosynthetic activities' instead...

Response: We have replaced "photosynthesis" by "photosynthetic parameters" (new line 302).

13. Suppl. Fig. S2 The figures in panels A and B seem too small. They might look more informative if appear as larger images one on top of the other.

Response: We have modified the Figure accordingly. It is now Supplemental Figure 5.

14. Translation efficiencies in tarnsplastomic tobacco lines: The data in Figure 8 and the data in Table 2 are not always in agreement, i.e., it is not clear why the number of significant sequences and translation efficiencies are not correlated. But I might of miss the sense of the MS data...

Response: We think that probably the reviewer meant the data in Figure 7 and in Table 2. To our opinion there are no major discrepancies in data. No AtpB was detected in *Nt*-IM13 plants. This mutant has albino phenotype (Figure 5B) and with this it resembles phenotype of *ΔatpB* knock-out mutant (Hager, 2002). Dual luciferase *E. coli* system revealed strong reduction in translation efficiency in pEK4-*Nt*-IM13, pEK4-*Nt*-IM16 and pEK4-*Nt*-IM18 (Figure 7). *Nt*-IM16 and *Nt*-IM18 have some green sectors on the leaves confirming minor yet detectible accumulation of AtpB.

15. discussion - check the sense of "...johansen Standard I-iota..".

Response: We have changed the wording (new lines 561-562).

Reviewer #2 (Comments for the Author):

The manuscript demonstrates the programmed ribosomal frameshift in chloroplast atpB gene especially in the design +2A mutant. While the analyses included necessary controls, the points below should be addressed before publication.

1) line 197: "no difference in the mobility of the protein band between the wild type and the I-iota was observed". -- I assumed that this statement refers to the lack of truncated protein in the mutant. Fig 2B only showed full length AtpB region. Please provides the western blot evidence for this statement (full blot image?).

Response: We have provided the blot membranes in Supplemental Figure 1A.

2) There are some inconsistencies of the names of the mutants in main text, figures, and tables. For example, line 286 used Nt-IM11 (AAG+/-0) vs Fig 5B used Nt-IM11 (AAG) vs Table 1 used Nt-IM11(AAG+/-0); Line 291 used Nt-IM13 (AAG+1) vs Fig 5B used IM13 (AAG+1A). Please make sure that they are consistent throughout the manuscript.

Response: We thank the reviewer for this comment. We have corrected the mutant names throughout the manuscript.

3) Table 2. It was not cleared what "No. of significant matches" and "No. of significant sequences" mean. Please include additional information in the legend. Eg. from what program? how significant defined?

Response: We have added the program name and a significance threshold in Table 2.

4) Figure 3 legend used the terms "b-" and "y-" is a bit odd and could be confused with negative ions. Most people use "b" and "y" or "b-ions" and "y-ions".

Response: We have modified the legend accordingly.

5) In figure for mass spectra result, eg. Fig 3D. "I-iota (+1A), +1" heading was used. The legend lacks explanation on what +1 after the comma mean. It wasn't until the main text mentioning Fig 8F that the reader would appreciate that this number identify the frameshift correction. It would be very informative and clearer to mention this in the figure legend.

Response: We thank reviewer for this comment and have included the explanation for the numbers in the legend for Figure 3D.

Below are typos that should be corrected

-line 268: "4B,)" should be "4B).

-line 315: "(AAG)]" should be "(AAG)".

-line 637: "aTyphoon" should be "a Typhoon".

-Fig 3 legend: "[Oe-Wt]" should be "[Oe-WT]"

-Fig 7: "pEK4-Nt-IM15 (+2A)" should be "pEK4-Nt-IM15 (AAG+2A)"

-Fig 9B and C: "Nt-IM11 (GAA)" should be "Nt-IM11 (AAG)"

Response: We have corrected the typos. Thanks for double checking.

Reviewer #3 (Comments for the Author):
This manuscript, which is well written, provides strong support for the operation of ribosomal frame shifting in plastids. A rigorous experimental approach provided a very good set of results. The identification of the phenomenon in Oenothera and the subsequent testing of the model in tobacco using appropriate constructs with variations in the polyA stretch was very good science. A comparison with E. coli further strengthens the paper.

Other points

1) The phenotype of the I-iota mutant plants is interesting. The mutant plastids are maintained in heteroplasmic plants allowing the sorting out of homoplasmic mutant sectors. The homogenous pale phenotype is understandable. The variegated phenotype is very interesting. Do the authors consider potential explanations such as dose-threshold models suggested, for example, for the Arabidopsis immutans phenotype (Rodermel). The tobacco -2A seedlings also have green sectors (Fig. 6C, 8B). Can these also be explained by a dose-threshold type model (Rodermel)? The authors may wish to include potential explanations of the variegated phenotype to help the reader.

Response: We thank reviewer for this suggestion. We have included the dose-threshold explanation in the discussion (new lines 473-482).

The photos suggest these are clear sectors with defined boundaries? Figure 1A and C (lower panel). The PCR analysis was on the clpP polymorphism. Can the possibility that some of the green sectors arise from slippage events during DNA replication that restore the atpB WT sequence be ruled out? The cDNA analyses in Fig. 10 argue against DNA slippage events and are consistent with the retention of mutant alleles in tobacco and Oenothera but were these conducted on the green or white sectors? A sentence addressing this issue of replication slippage could be included.

Response: We thank reviewer for this valuable comment. Indeed, we have performed the cDNA analysis on leaves of different developmental stages of I-iota (mature, intermediate, and young). Also, here no indication for transcriptional slippage was detected. We have included these data in Supplemental Figure 4.

2) The amino acid sequences and DNA sequences were presented separately.
To facilitate readability, the DNA sequence with the predicted translation products for the various frameshifts could be placed in the main text for I-iota (+1A) In Fig 1D.

Response: We have included the amino acid sequence in Figure 1D.

3) Fig. 2B. The first set of 50 and 100% lanes (mature) under the I-iota (+ 1A) appear to show accumulation of AtpB equivalent to the WT lanes. The main text suggests mature I-iota (+ 1A) leaves accumulate atpB to higher levels than immature leaves. If the accumulation is to WT levels this could be mentioned in the main text.

Response: We have performed new western blot analysis using leaf crude extracts. As the AtpB antibody from Agrisera cross react with mito AtpB, we, therefore, probed the membranes with antibodies recognized AtpA and AtpB from the Berzborn collection (Figure 2B). The abundance of AtpA/B is increased from young to mature leaves in I-iota, but still lower compared to the wild type. Please also see response to Reviewer 1's comment 5.

4) Abstract: 'We further show that the homopolymeric composition... is essential for recoding. Plants.... DISRUPTED oligo A stretch have an albino phenotype.
This meaning of this sentence wasn't clear. If this means replacement of Lysine AAA codons by AAG codons this specific detail would clarify the sentence.

Response: We have modified this part of the abstract accordingly (new line 41).

5) Fig. 5E. The text indicates identical peptides were detected in WT and Nt-IM12(+1A) tissues. Fig 5E only shows one LC MS/MS spectrum. If the WT spectrum was not included this could be mentioned in the main text. Consider including the amino acids above the DNA sequences in Fig. 5A so the reading frame and presence of only two lysines in tobacco is clear.

Response: We have included the *Nt*-WT LC MS/MS spectrum in Supplemental Figure 3.

6) Fig. 3D. The y14 peak is not labeled.

Response: We have labeled the y14 in Figure 3D.

7) pg14 line 404. 'A peptide resulting from +3 correction was detected in Nt-IMI16(-1A) (Figure 5E)'. The sentence could be clearer by indicating the expected number of lysines for the +3 correction.

Response: We have included the missing information of detected lysine stretch (two lysine) and corrected the typo in the main text: exchanged error "+2" by correct "-1" (new lines 409-410).

8) Figure 1 legend. Typo 'homoplasmy '

Response: We have corrected the typo.

9) Fig. 10 legend Typo 'ther'

Response: We have exchanged "ther" by "the".

10) Pg 18 line 522. Typo Ivested (investigated)

Response: We have corrected this typo (new line 538).

TPC2020-RA-00686R1 2nd Editorial decision – *acceptance pending* **Dec. 31, 2020**

We are pleased to inform you that your paper entitled "Correction of frameshift mutations in the atpB gene by translational recoding in chloroplasts of Oenothera and tobacco." has been accepted for publication in The Plant Cell, pending a final minor editorial review by journal staff. At this stage, your manuscript will be evaluated by a Science Editor with respect to its presentation of scientific content, compliance with journal policies, and presentation for a broad readership.

Final acceptance from Science Editor **Feb. 2, 2021**
